# Injectable ROS-scavenging and NIR-responsive nanocomposite hydrogel for staphylococcus aureus-infected diabetic wound healing

**DOI:** 10.1186/s12951-026-04306-4

**Published:** 2026-03-27

**Authors:** Ming Cai, Zhao Liu, Xun Sun, Ziming Liao, Yu Gou, Xuan Yang, Ying Qi, Guosheng Xing, Tingting Liu, Wenjun Zhao, Xiaoyuan Duan, Tao Zhang, Bin Yao, Weiguo Xu

**Affiliations:** 1 Clinical College of Orthopedics, Tianjin Medical University, Tianjin, 300070 PR China; 2 Hebei Key Laboratory of Systems Biology and Gene Regulation, Department of Orthopedics, First Affiliated Hospital of Hebei North University, Zhangjiakou, 075000 Hebei PR China; 3 Tianjin Hospital, Tianjin University, Tianjin, 300211 PR China; 4 Academy of Medical Engineering and Translational Medicine, Tianjin University, Tianjin, 300072 PR China; 5 The Orthopaedic Institute, Tianjin Hospital, Tianjin, 300050 PR China; 6 Department of Laboratory Diagnosis, The 971th Hospital, Qingdao, 266072 PR China

**Keywords:** Diabetic wound infection, injectable hydrogel, nanocomposite, antibacterial activity, oxidative microenvironment regulation.

## Abstract

Chronic diabetic wounds are often complicated by persistent infections and oxidative stress, which impede effective healing. In this study, we developed an injectable, dual-responsive nanocomposite hydrogel designed to simultaneously scavenge reactive oxygen species (ROS) and deliver photothermal and photodynamic therapy for wound treatment. The hydrogel was synthesized via dynamic boronate-ester crosslinking of poly(vinyl alcohol) (PVA) with a bis-boronic acid linker (TSPBA), enabling efficient ROS quenching. Incorporation of palygorskite/molybdenum disulfide (PGS/MoS₂) nanocomposites imparted photothermal and photodynamic properties, activated by NIR light. In vitro tests demonstrated that the PVA-TSPBA@PGS/MoS₂ hydrogel effectively scavenged ROS, induced mild hyperthermia (~ 48 °C) under NIR, and exhibited significant antibacterial activity against Staphylococcus aureus. In vivo, the hydrogel accelerated wound closure in a diabetic mouse model, with a 46.7% residual area at day 7, compared to higher residual areas in control groups (66.5–92.4%). By day 14, nearly complete wound healing was achieved, accompanied by reduced bacterial load and inflammatory markers, as well as enhanced re-epithelialization, angiogenesis, and collagen remodeling. These findings suggest that this multifunctional hydrogel represents a promising strategy for the treatment of infected diabetic wounds, offering a controlled, localized approach to wound healing.

**Graphical Abstract:**

**Figure:**
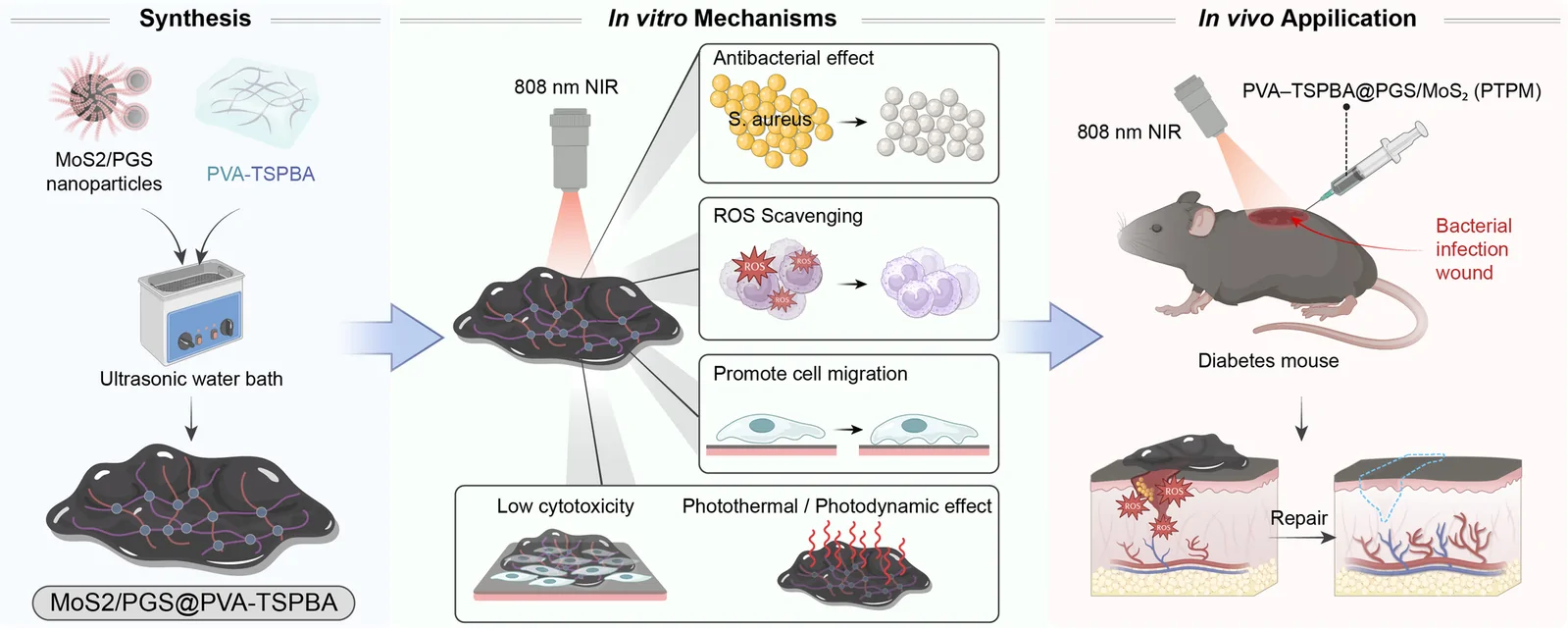

**Supplementary Information:**

The online version contains supplementary material available at https://doi.org/10.1186/s12951-026-04306-4.

## Introduction

 Chronic wounds are a prevalent and challenging complication of diabetes mellitus. Their non-healing nature significantly impairs patients’ quality of life, heightens the risk of infection and amputation, and imposes a substantial burden on healthcare systems [1–3]. These wounds present a dysregulated microenvironment where hyperglycemia, persistent bacterial colonization, oxidative stress, ischemia, hypoxia, and chronic inflammation collectively disrupt angiogenesis, re-epithelialization, and extracellular matrix (ECM) remodeling, ultimately hindering wound closure [4–6]. Conventional interventions, including systemic antibiotics and commercial wound dressings, can partially control infection and maintain a moist healing environment; however, they are insufficient to regulate reactive oxygen species (ROS), or simultaneously promote vascular and epithelial regeneration [7–11]. Accordingly, the development of intelligent, multi-responsive wound dressings capable of on-demand regulation and multi-target synergy to mitigate infection and oxidative stress while reconstructing a pro-regenerative microenvironment has emerged as a critical priority in diabetic wound management.

Owing to their high water content, excellent biocompatibility, and inherent adhesiveness, hydrogels are commonly used in chronic wound management but often fail to meet the multifunctional requirements for diabetic wound therapy, such as ROS scavenging, antibacterial effects, angiogenesis, and epithelial regeneration [12–15]. Incorporation of nanomaterials provides an effective strategy to overcome these limitations. Metallic and transition-metal nanomaterials, such as silver, copper, zinc, and molybdenum disulfide (MoS₂), can disrupt bacterial membranes, interfere with metabolic pathways, and modulate the wound microenvironment, exhibiting potent antibacterial and tissue-repairing properties [16–18]. Moreover, many nanomaterials possess intrinsic abilities to regulate oxidative stress and enhance angiogenesis, supporting tissue regeneration [11, 19, 20]. When integrated with hydrogel matrices, these nanomaterials enable the fabrication of functional nanocomposite hydrogels that achieve coordinated antibacterial, anti-inflammatory, and pro-regenerative effects [20–22].

**Fig. 1:**
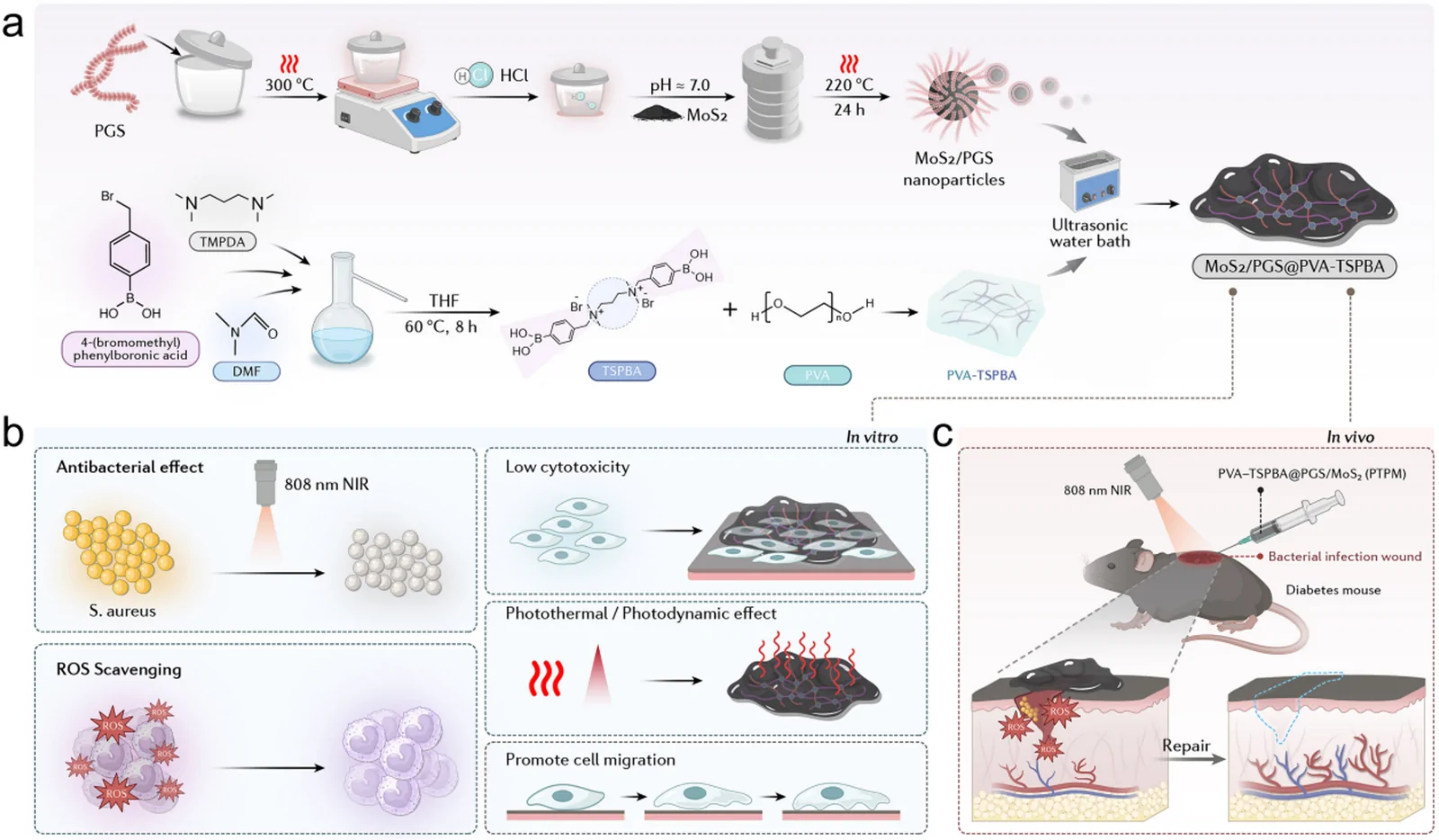
Schematic overview of the preparation and therapeutic functions of the PVA-TSPBA@PGS/MoS₂ hydrogel. **a**) Formation of the PVA-TSPBA hydrogel through dynamic boronate ester crosslinking and incorporation of PGS/MoS₂ nanocomposites. **b**) Multifunctional characteristics of the resulting hydrogel, including robust antibacterial activity, ROS scavenging, low cytotoxicity, photothermal and photodynamic effects, and enhanced cell migration. **c**) Synergistic therapeutic mechanism by which the PVA-TSPBA@PGS/MoS₂ hydrogel promotes diabetic infected wound repair through coordinated antibacterial, anti-inflammatory, antioxidative, and pro-regenerative actions.PVA, poly(vinyl alcohol); TSPBA, N¹-(4-boronobenzyl)-N³-(4-boronophenyl)-N¹,N¹,N³,N³-tetramethylpropane-1,3-diamine; PGS, Palygorskite; MoS₂, molybdenum disulfide; ROS, reactive oxygen species

We developed a dual-responsive inorganic–organic hybrid hydrogel designed to modulate the oxidative microenvironment and provide photothermal and photodynamic antibacterial activity. Testing against antibiotic-resistant strains (e.g., MRSA) will be an important next step. The hydrogel formed rapidly through dynamic boronate-ester crosslinking between poly(vinyl alcohol) (PVA) and a bisboronic-acid linker, N¹-(4-boronobenzyl)-N³-(4-boronophenyl)-N¹,N¹,N³,N³-tetramethylpropane-1,3-diamine (TSPBA), conferring ROS scavenging and free-radical neutralization capabilities. In parallel, the incorporation of Palygorskite (PGS)/MoS₂ self-assembled nanocomposites introduced near-infrared (NIR)-triggered photothermal and photodynamic effects. The needle-like PGS morphology [23, 24] enhanced bacterial membrane disruption and increased the specific surface area and light-utilization efficiency of MoS₂, strengthening antibacterial and anti-inflammatory performance while maintaining favorable biocompatibility. Infected diabetic wounds are characterized by co-existing bacterial burden and oxidative stress, which jointly sustain inflammation and impair angiogenesis and re-epithelialization. Therefore, we selected (i) a boronate-ester dynamic network to scavenge ROS and modulate the oxidative microenvironment, and (ii) nanocomposites to provide local antibacterial action, with optional NIR-triggered photothermal/ROS generation for on-demand enhancement [22, 25, 26]. The PVA-TSPBA@PGS/MoS₂ (PTPM) hydrogel represents a controllable, multifunctional, and translational platform for precision therapy in diabetic wound infections (Fig. 1).

## Results and discussion

### Design and assembly of the PTPM smart composite hydrogel

To validate the design concept, a dual-responsive inorganic–organic hybrid hydrogel (PVA-TSPBA@PGS/MoS₂) was fabricated. The system comprises a TSPBA-modified PVA network functioning as a matrix for inorganic nanocomposites, whereas PGS/MoS₂ acts as the photothermal-photodynamic functional component. PGS/MoS₂ nanocomposites are physically entrapped within the porous hydrogel network (supported by SEM mapping), which is expected to favor local retention at the wound site. The preparation and optimization process included the following steps:

#### Synthesis and characterization of TSPBA

The ROS-responsive linker, TSPBA, was successfully synthesized and verified through both solid-state and solution-state ¹H nuclear magnetic resonance (¹H NMR) spectroscopy. The characteristic proton distribution, chemical shifts, and coupling patterns confirmed successful formation of the target molecular structure. (Figures S1–S2) [27].

#### Optimization of the PVA-TSPBA hydrogel composition

Initially, 10%, 15%, 20%, 25%, and 30% PVA (w/v) hydrogels were prepared, which demonstrated adequate injectability, although gelation behavior varied significantly, including instances of non-gelation, 56.6, 26.3, 12.8, and 5.3 min, respectively (Figures S3 and S4). Considering that subsequent incorporation of TSPBA and PGS/MoS₂ was expected to accelerate gelation and potentially affect injectability, PVA concentrations of 10%, 15%, and 20% were selected for further evaluation.

These three PVA concentrations were crosslinked with 5%, 10%, and 15% TSPBA (w/v) solutions, generating nine PVA-TSPBA formulations(10%PVA-5%TSPBA,10%PVA-10%TSPBA,10%PVA-15%TSPBA,15%PVA-5%TSPBA,15%PVA-10%TSPBA,15%PVA-15%TSPBA,20%PVA-5%TSPBA,20%PVA-10%TSPBA and 20%PVA-15%TSPBA). The resulting gelation times were 66.4, 46.1, 38.9, 33.8, 21.6, 16.8, 8.6, 3.5, and 2.3 min, respectively. Based on feasibility for injection and in situ gel formation, three formulations, including 10%PVA–10%TSPBA, 10%PVA–15%TSPBA, and 15%PVA–5%TSPBA, were shortlisted (Figure S5).

Following incorporation of PGS/MoS₂ nanocomposites, gelation kinetics were reassessed and recorded as 35.9, 28.9, and 19.4 min, respectively, demonstrating further reductions in gelation time. Considering the operative window required for in vivo administration, the 15%PVA–5%TSPBA formulation was identified as the most suitable (Figure S6). Photodynamic assays using methylene blue (MB) and 1,3-diphenylisobenzofuran (DPBF) probes confirmed that this composition exhibited the highest NIR-induced ROS generation (Figure S7); therefore, it was selected as the optimal hydrogel matrix.

#### Optimization of the PGS/MoS₂ nanocomposite ratio

PGS was acid- and heat-treated prior to hydrothermal synthesis with varying mass ratios of PGS (2.5%, 5%, 7.5%, and 10%) to MoS₂. Scanning electron microscopy (SEM) revealed a uniform porous morphology, and elemental mapping confirmed homogeneous distribution of silicon (Si), oxygen (O), molybdenum (Mo), and sulfur (S) across all formulations (Figures S8–S10). X-ray diffraction (XRD) patterns verified successful hybridization of the PGS/MoS₂ structures (Figure S11). Photothermal conversion under NIR irradiation demonstrated that the 7.5% PGS/92.5% MoS₂ nanocomposite achieved the highest temperature (72.2 °C), indicating superior photothermal efficiency (Figure S12). Consistently, MB and DPBF photodynamic assays confirmed that this composition generated the highest reactive ROS yield under NIR exposure (Figure S13). Consequently, the 7.5% PGS/92.5% MoS₂ ratio was selected for the final formulation. Therefore, the optimized hybrid hydrogel was established as 15% PVA–5% TSPBA@7.5% PGS/92.5% MoS₂.

### Characterization of the PTPM smart composite hydrogel

**Fig. 2:**
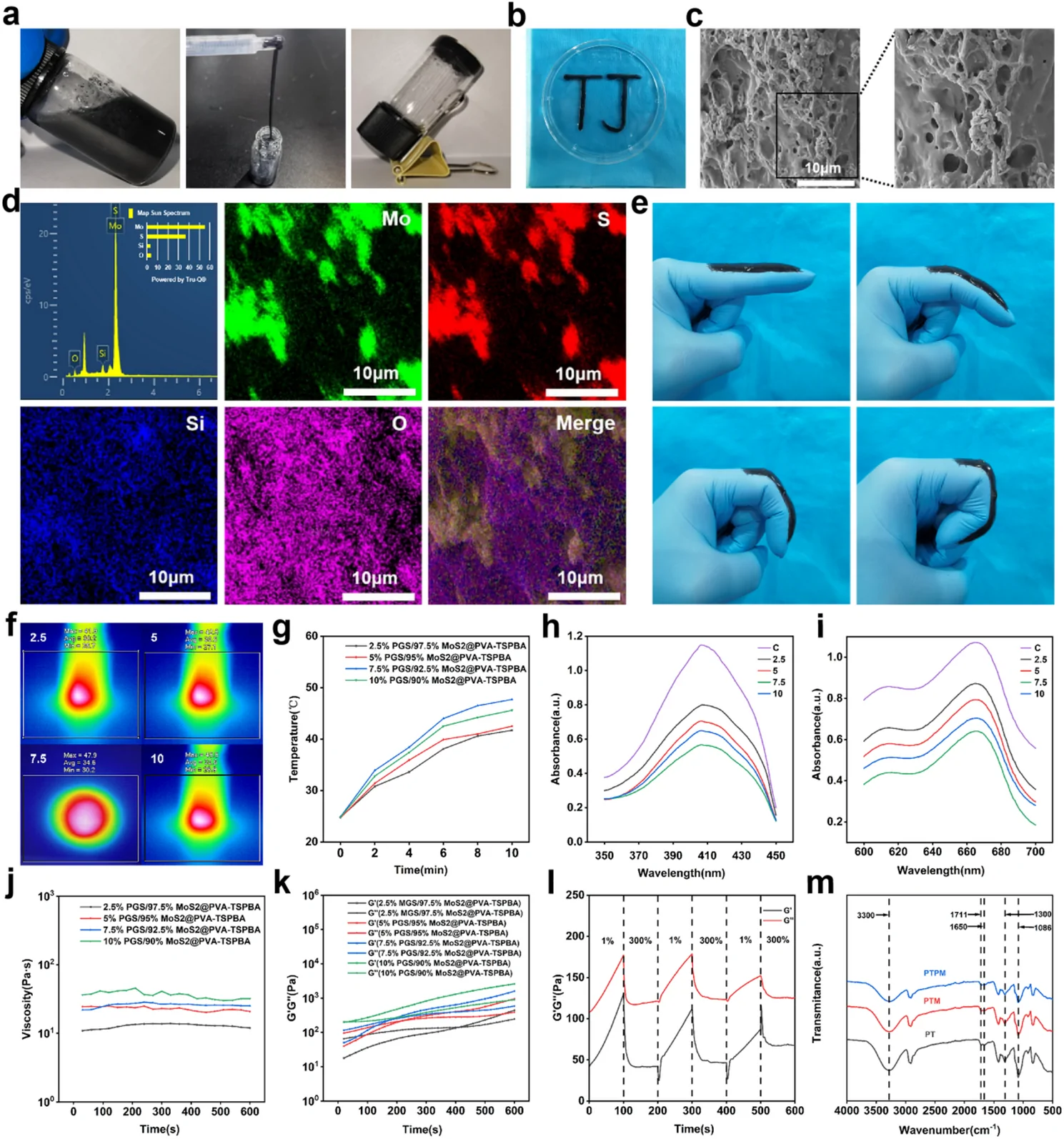
Characterization of the PVA-TSPBA@7.5%PGS/92.5%MoS₂ hydrogel. **a**) Gelation testing verified successful hydrogel formation of the PVA-TSPBA@7.5%PGS/92.5%MoS₂ hydrogel. b) Injectability evaluation confirmed excellent syringeability and rapid in situ gelation. **c**) SEM demonstrated a highly porous microarchitecture of the PVA-TSPBA@7.5%PGS/92.5%MoS₂ hydrogel. **d**) Elemental mapping (Mo, S, Si, and O) revealed uniform distribution of PGS/MoS₂ nanocomposites within the hydrogel matrix. **e**) Mechanical elasticity and joint adaptability testing revealed superior flexibility, adhesion, and conformability during joint motion. **f**,** g**) Photothermal evaluation under NIR (808 nm,1.0 W·cm⁻², 10 min) irradiation demonstrated safe and efficient photothermal conversion for PVA-TSPBA@7.5%PGS/92.5%MoS₂, supported by temperature-elevation and time–temperature curves (2.5, 5, 7.5, and 10 correspond to PVA-TSPBA@2.5%PGS/97.5%MoS₂, PVA-TSPBA@5%PGS/95%MoS₂, PVA-TSPBA@7.5%PGS/92.5%MoS₂, and PVA-TSPBA@10%PGS/90%MoS₂ hydrogels, respectively). **h**, **i**) Photodynamic assessment revealed maximal ROS generation under NIR (808 nm, 1.0 W·cm⁻², 10 min) irradiation for the PVA-TSPBA@7.5%PGS/92.5%MoS₂ formulation, as measured using DPBF and MB. (C, 2.5, 5, 7.5, and 10 correspond to control, PVA-TSPBA@2.5%PGS/97.5%MoS₂, PVA-TSPBA@5%PGS/95%MoS₂, PVA-TSPBA@7.5%PGS/92.5%MoS₂, and PVA-TSPBA@10%PGS/90%MoS₂ hydrogels, respectively). **j**) Rheological testing confirmed suitable viscosity for practical handling and injection. **k**) Time-dependent rheological curves for storage and loss moduli (G′ and G″) indicated a balanced sol–gel transition profile. **l**) strain-dependent testing demonstrated reversible mechanical behavior under low and high shear stress, supporting excellent injectability and structural integrity. **m**) Fourier-transform infrared spectroscopy validated successful formation of the PTPM, PTM, and PT hydrogels.SEM, scanning electron microscopy; NIR, near-infrared; ROS, reactive oxygen species; PVA, polyvinyl alcohol; TSPBA, N¹-(4-boronobenzyl)-N³-(4-boronophenyl)-N¹,N¹,N³,N³-tetramethylpropane-1,3-diamine; PGS, Palygorskite; MoS₂, molybdenum disulfide; PT, PVA-TSPBA hydrogel; PTM, PVA-TSPBA@MoS₂ hydrogel; PTPM, PVA-TSPBA@PGS/MoS₂ hydrogel

The as-prepared PTPM hydrogel demonstrated excellent injectability in its initial sol state and gradually transitioned into a cohesive, adhesive gel (Fig. 2a, b; Figures S14–S16). Our hydrogel is unique in that it is injectable, forms in situ, and responds to ROS, making it a localized platform that can regulate the microenvironment, especially in the presence of both infection and oxidative stress. Additionally, it offers the option for light-triggered enhancement. In contrast, commercial PVA/Ag dressings are typically pre-made, easy to use, and primarily exert antibacterial effects through silver. Furthermore, a direct comparison with commercial dressings was not conducted in this study, but this represents an important direction for future research. SEM revealed a uniform porous microstructure, with elemental mapping confirming homogeneous distribution of Mo, S, Si, and O, indicative of successful PGS/MoS₂ hybridization within the hydrogel matrix (Fig. 2c, d). When applied to a human fingertip, the hydrogel exhibited robust adhesion and elasticity, conforming to joint movement without cracking or detachment (Fig. 2e).

Using the optimized PVA-TSPBA carrier, we further evaluated hydrogels incorporating 2.5%, 5%, 7.5%, and 10% PGS/MoS₂ nanocomposites. Photothermal analysis revealed that the PVA-TSPBA@7.5% PGS/92.5% MoS₂ formulation exhibited superior light-to-heat conversion efficiency compared to other formulations, achieving a maximum temperature of 47.90 °C. This value is safely below the 50 °C threshold, ensuring biocompatibility for both wound and in vivo applications(Fig. 2f, g) [28]. Zhao successfully developed a multifunctional hydrogel (rGB/QCS/PDA-PAM) dressing, which exhibited high antibacterial activity against MRSA at a mild temperature of 49.6 °C [28]. Yin developed a novel photothermal agent, PMI-NOP, and both in vitro and in vivo experiments demonstrated that mild photothermal treatment at 45 °C could achieve effective antibacterial effects [29].

Photodynamic analysis, using methylene blue (MB) and 1,3-diphenylisobenzofuran (DPBF) assays, confirmed that the 7.5% PGS/92.5% MoS₂ formulation generated the highest levels of reactive oxygen species (ROS) under NIR irradiation (Figs. 2h, i). Rheological characterization showed stable viscosity post-gelation for all formulations, with the 7.5% PGS/92.5% MoS₂ hydrogel demonstrating an optimal balance of viscoelastic properties (Fig. 2j). The sol–gel transition occurred gradually, supporting favorable injectability and mechanical stability (Fig. 2k).

These findings indicate that PVA-TSPBA@7.5% PGS/92.5% MoS₂ possesses the most desirable rheological and mechanical properties, including excellent injectability during administration, gradual gelation with stable elasticity, and strong adhesion after solidification. Its consistent performance under low- and high-shear conditions further emphasizes its structural robustness (Fig. 2l).

Fourier-transform infrared (FTIR) spectroscopy confirmed successful synthesis of the organic components within the hybrid system. Characteristic absorption bands corresponding to hydroxyl (–OH, 3300 cm⁻¹), carbonyl (C = O, 1711 cm⁻¹), aromatic ring (1650 cm⁻¹), C–O (1300 cm⁻¹), and C–H (1086 cm⁻¹) stretching vibrations were observed in PTPM, PVA-TSPBA@MoS₂, and PVA-TSPBA hydrogels, verifying the formation of the PVA-TSPBA network (Fig. 2m).

### Biological functionality and biocompatibility of PTPM hydrogel

**Fig. 3:**
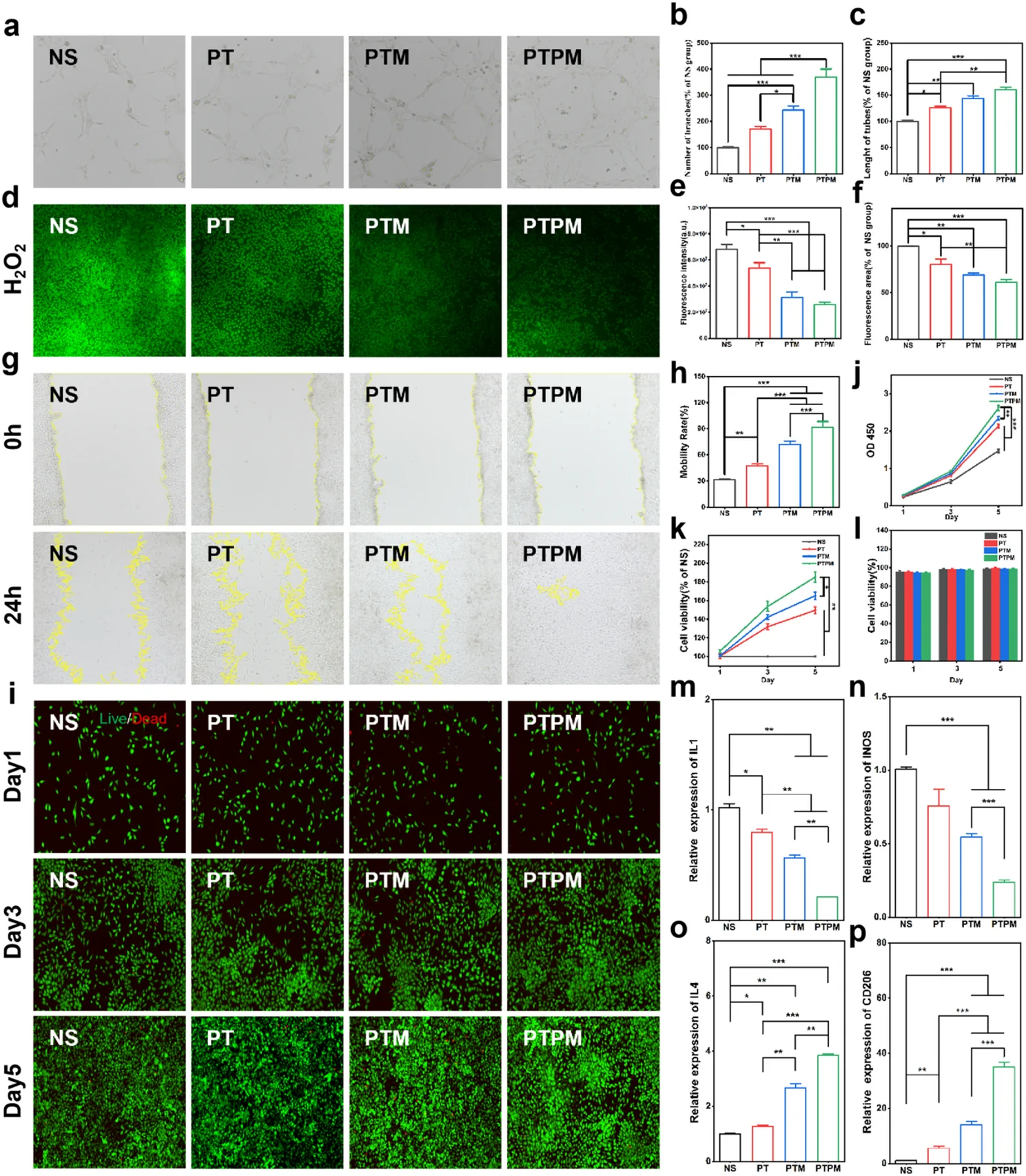
Biological functionality and biocompatibility of the PTPM hydrogel. **a**) Tube formation assay in HUVECs, demonstrating the strongest proangiogenic activity in the PTPM hydrogel group. **b**,** c**) Quantitative analyses of branch number and total tube length further confirmed its superior vascularization potential(*n* = 3 per group). **d**) Intracellular ROS levels in L929 fibroblasts cultured with different hydrogels revealed the most efficient ROS scavenging in the PTPM group, **e**,** f**) supported by quantitative fluorescence intensity and area measurements. **g**) Scratch-wound assays at 0 and 24 h indicated markedly enhanced cell migration with PTPM treatment(*n* = 3 per group), **h**) corroborated by statistical analysis of migration rates(*n* = 3 per group). **i**) Live/dead fluorescence staining of L929 cells on days 1, 3 and 5 showed excellent cytocompatibility across all groups, with the PTPM hydrogel promoting the greatest proliferation over time. **j–l**) Quantitative assessments of fluorescence intensity, viability, and proliferation on days 1, 3 and 5 further verified enhanced cellular growth and biocompatibility in the PTPM group(*n* = 3 per group). **m–p**) qPCR analysis of proinflammatory (IL-1, iNOS) and anti-inflammatory markers (IL-4, CD206) demonstrated that the PTPM hydrogel most effectively suppressed inflammatory responses and promoted an anti-inflammatory phenotype(*n* = 3 per group). All data are presented as mean ± SD; *P < 0.05, **P < 0.01, ***P < 0.001.HUVECs, human umbilical vein endothelial cells; ROS, reactive oxygen species; qPCR, quantitative polymerase chain reaction; NS, normal saline; PVA, polyvinyl alcohol; TSPBA, N¹-(4-boronobenzyl)-N³-(4-boronophenyl)-N¹,N¹,N³,N³-tetramethylpropane-1,3-diamine; PGS, Palygorskite; MoS₂, molybdenum disulfide; PT, PVA-TSPBA hydrogel; PTM, PVA-TSPBA@MoS₂ hydrogel; PTPM, PVA-TSPBA@PGS/MoS₂ hydrogel

To assess the biological functionality of the PTPM hydrogel, an in vitro angiogenesis assay was performed using human umbilical vein endothelial cells (HUVECs). The PTPM hydrogel demonstrated substantially enhanced proangiogenic activity compared with normal saline (NS), PVA-TSPBA (PT), and PVA-TSPBA@MoS₂ (PTM) hydrogel groups (Fig. 3a). Quantitative analysis showed that HUVECs treated with the PTPM hydrogel formed significantly more branch points and longer tubular structures, indicating superior angiogenic capability (Fig. 3b, c).

To evaluate further the ROS scavenging capacity of the hydrogel at the cellular level, L929 fibroblasts were exposed to hydrogen peroxide to induce oxidative stress and subsequently stained with 2’,7’-dichlorofluorescein diacetate (DCFH-DA); green fluorescence reflected intracellular ROS levels. Untreated cells did not fluoresce, whereas cells treated with the PT hydrogel exhibited strong fluorescence, and those treated with the PTM hydrogel demonstrated weaker signals. Notably, fluorescence was almost abolished in the PTPM group, indicating effective ROS elimination (Fig. 3d, Figure S17). Quantitative fluorescence analysis confirmed a pronounced reduction in fluorescence intensity and area, demonstrating a substantial decrease in intracellular ROS levels (Fig. 3e, f).

A scratch assay was performed to assess cell migration. Artificial wounds were created in confluent L929 fibroblast monolayers, and cell migration into the wound areas was evaluated at 0 and 24 h. At 24 h, the cells treated with the PTPM hydrogel exhibited the greatest wound closure, indicating enhanced migration (Fig. 3g). Quantitative analysis further confirmed the superior pro-migratory effect of the PTPM hydrogel (Fig. 3h).

Live/dead fluorescence staining was performed to assess cytocompatibility over 1, 3, and 5 days. All groups demonstrated increased live cell numbers over time, with the PTPM hydrogel exhibiting the highest proliferation rate (Fig. 3i–k). Under all conditions, the proportion of viable cells exceeded 90% without significant differences among groups, indicating excellent cytocompatibility and supporting the hydrogel’s suitability for biomedical applications (Fig. 3i, l).

To evaluate anti-inflammatory properties, an L929 inflammation model was induced with lipopolysaccharide (LPS) and treated with the respective hydrogels, followed by quantitative polymerase chain reaction (qPCR) analysis. In the PTPM hydrogel group, expression of proinflammatory genes (IL-1 and iNOS) was significantly downregulated, whereas anti-inflammatory gene expression (IL-4 and CD206) was significantly upregulated compared to the NS, PT, and PTM groups (Fig. 3m–p). These findings indicate that the PTPM hydrogel effectively attenuated inflammatory responses and exhibited the most potent anti-inflammatory activity among the tested formulations.

### In vitro antibacterial performance of the PTPM hydrogel

#### Bactericidal activity

**Fig. 4:**
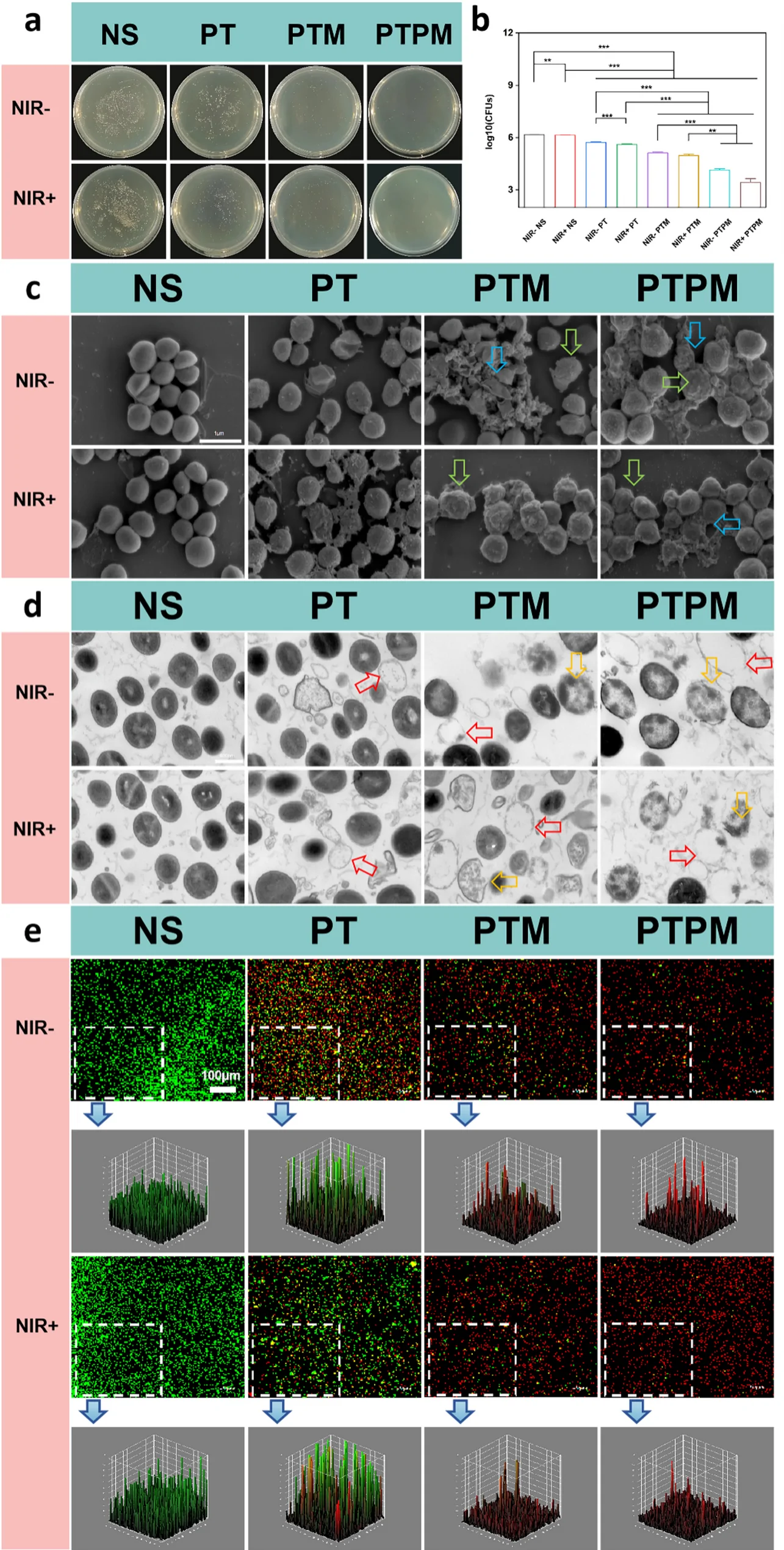
In vitro antibacterial performance of PTPM hydrogels. **a**) Representative agar plate images exhibit *Staphylococcus aureus* colonies following treatment with NS, PT, PTM, and PTPM hydrogels. “NIR (−/+)” indicates the absence or presence of 808 nm laser irradiation (1.0 W·cm⁻², 10 min). The NIR+PTPM group exhibited the strongest antibacterial effect. **b**) The remaining quantitative colony-forming unit (log10(CFUs)) counts further confirmed that the NIR+irradiated PTPM hydrogels exhibited the most significant antibacterial effect(*n* = 3 per group). Data are presented as mean ± SD; *P < 0.05, **P < 0.01, ***P < 0.001. **c**) Representative SEM images show bacterial morphology after different treatments. Green arrows denote collapsed cell membranes, and blue arrows indicate ruptured membranes (scale bar, 1 μm), with the most extensive disruption in the NIR+PTPM group. **d**) Representative TEM images illustrate intracellular structural damage. Yellow arrows indicate partial membrane rupture, while red arrows mark bacterial debris and complete membrane fragmentation (scale bar, 1 μm). Pronounced cytoplasmic leakage and cell collapse are seen in the NIR+PTPM group. **e**) Live/dead fluorescence staining demonstrated bacterial viability, with SYTO9 labeling live bacteria (green) and PI labeling dead bacteria (red) (scale bar, 100 μm). The NIR+PTPM hydrogel induced the highest bacterial mortality.NS, normal saline; PVA, polyvinyl alcohol; TSPBA, N¹-(4-boronobenzyl)-N³-(4-boronophenyl)-N¹,N¹,N³,N³-tetramethylpropane-1,3-diamine; PGS, Palygorskite; MoS₂, molybdenum disulfide; PT, PVA-TSPBA hydrogel; PTM, PVA-TSPBA@MoS₂ hydrogel; PTPM, PVA-TSPBA@PGS/MoS₂ hydrogel; NIR, near-infrared; SEM, scanning electron microscopy; TEM, transmission electron microscopy; PI, propidium iodide; SYTO9, nucleic acid live-cell stain

The antibacterial activity of the PTPM hydrogel was assessed against *Staphylococcus aureus* (**the antibacterial activity in this study was limited to** ***Staphylococcus aureus***, **a Gram-positive bacterium**,** and further validation against Gram-negative pathogens**,** such as** ***Pseudomonas aeruginosa***, **as well as biofilm models**,** will be important directions for future research** ) under NIR (808 nm,1.0 W·cm⁻², 10 min) irradiation and non-irradiated conditions using a plate colony-counting assay. Bacterial suspensions were incubated with each hydrogel formulation, followed by NIR laser exposure for a predetermined duration, or maintenance in the dark. Next, the mixtures were plated on Luria-Bertani (LB) agar and incubated overnight. The PTPM hydrogel demonstrated the strongest bactericidal effect under NIR irradiation, with a substantial reduction in colony formation compared to all other groups (Fig. 4a and b).

#### Bacterial membrane disruption

To investigate the antibacterial mechanism, the morphological integrity of *S. aureus* was evaluated using SEM. In both NIR-irradiated and non-irradiated NS groups, bacterial cells retained smooth, intact surfaces. In the NIR and non-NIR PT groups, occasional deformation and surface wrinkling were observed. In contrast, the NIR and non-NIR PTM and PTPM groups exhibited extensive and irreversible membrane disruption, including rupture, distortion, shrinkage, and collapse, as indicated by green arrows (collapsed cell walls) and blue arrows (ruptured or fragmented membranes) (Fig. 4c). The NIR+PTPM group demonstrated the most pronounced structural destruction.

Transmission electron microscopy further confirmed severe intracellular damage. In the PTM and PTPM groups, with or without NIR exposure, cytoplasmic contents appeared dispersed and detached from the membrane, consistent with membrane rupture and leakage of intracellular components. Yellow arrows denote partial wall rupture, while red arrows indicate complete structural disintegration and residual bacterial debris (Fig. 4d). Consistently, the NIR+PTPM group exhibited the greatest bactericidal effect.

#### Live/dead bacterial staining

To validate the antibacterial efficacy, a live/dead co-staining assay using SYTO9 (green, viable cells) and propidium iodide (PI; red, non-viable cells) was conducted. The NIR+PTPM group exhibited the highest proportion of red fluorescence, reflecting extensive bacterial death and minimal survival (Figs. 4e and S18). These findings confirm that the PTPM hydrogel, particularly under NIR irradiation, exerted synergistic photothermal and photodynamic antibacterial activity with superior efficacy against *S. aureus*.

### In vivo evaluation of PTPM hydrogel in full-thickness diabetic wound healing

**Fig. 5:**
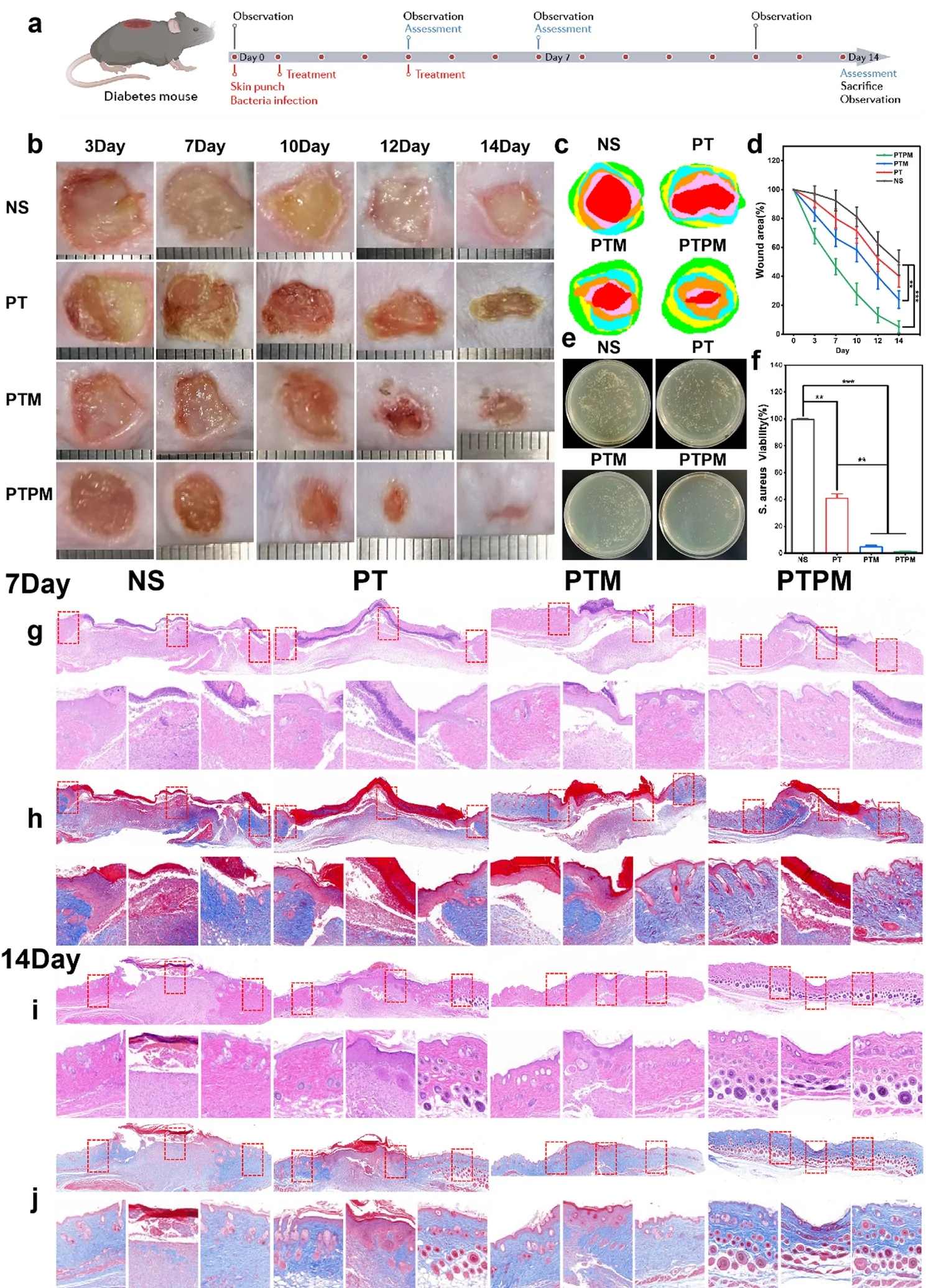
In vivo antibacterial efficacy and wound-healing performance of PTPM hydrogel in *S. aureus*–infected diabetic mice. **a**) Schematic illustration of the *S. aureus*–infected diabetic wound model, treatment regimen, and therapeutic procedure. **b**) Representative photographs of infected full-thickness wounds on day 3, 7, 10, 12, and 14 post-treatment. The PTPM hydrogel group exhibited the most efficient wound closure among all groups. Additionally, 808 nm NIR light was used to activate the photothermal and photodynamic effects on the hydrogel (or normal saline), with a power density of 1.0 W·cm⁻² for 10 min. **c**) Wound-healing trajectory mapping of diabetic wounds on days 0, 3, 7, 10, 12, and 14, where green, yellow, blue, orange, pink, and red indicate the wound areas at each corresponding time point. The PTPM group demonstrated the fastest wound contraction. **d**) Quantitative analysis of relative wound areas from day 0 to day 14. PTPM hydrogel significantly accelerated wound closure compared to other groups. (*n* = 3 per group) Data are presented as mean ± SD; *P < 0.05, **P < 0.01, ***P < 0.001. **e**) Representative photographs of bacterial colonies cultured from wound exudates collected on day 3, showing the strongest antibacterial effect in the PTPM group. **f**) Quantitative analysis of bacterial survival in infected wound tissues, confirming the superior antibacterial efficacy of PTPM hydrogel.(*n* = 3 per group) Data are presented as mean ± SD; *P < 0.05, **P < 0.01, ***P < 0.001. **g**) Representative H&E–stained sections and magnified images of wound tissues on day 7. The PTPM group displayed enhanced re-epithelialization with continuous epithelial bridging, reduced necrosis and scab formation, diminished inflammatory infiltration and edema, and dense, uniform granulation tissue, indicating a well-progressed transition from the inflammatory to proliferative phase. **h**) Representative MT-stained sections and enlarged views of wound tissues on day 7. The PTPM group exhibited increased collagen deposition with continuous, aligned fibers, whereas other groups showed loose, discontinuous, or unevenly stained collagen bundles. **i**) Representative H&E-stained sections and magnified images of wound tissues on day 14. The PTPM group showed nearly complete and continuous re-epithelialization, with epidermal thickness and architecture resembling normal skin, minimal inflammatory cell infiltration, and mature granulation tissue with abundant neovascularization and aligned fibroblasts. **j**) Representative MT-stained sections and enlarged images of wound tissues on day 14. The PTPM group demonstrated denser, more continuous collagen deposition with thicker, parallel bundles, indicating advanced ECM remodeling, whereas other groups exhibited sparse, disorganized, or fragmented fibers.*S. aureus*, *Staphylococcus aureus*; H&E, hematoxylin–eosin; MT, Masson’s trichrome; ECM, extracellular matrix

Considering the potent antibacterial properties and multifunctional bioactivity of the PTPM hydrogel in vitro, we evaluated its in vivo antibacterial and wound-healing performance in a full-thickness *S. aureus*–infected diabetic mouse model (**the antibacterial activity in this study was limited to** ***Staphylococcus aureus***, **a Gram-positive bacterium**,** and further validation against Gram-negative pathogens**,** such as** ***Pseudomonas aeruginosa***, **as well as biofilm models**,** will be important directions for future research**) (Fig. 5a).

Diabetic mice (specific pathogen-free) were anesthetized, and a 6-mm circular full-thickness excisional wound was created on the dorsal skin. An *S. aureus* suspension was applied to the wound and incubated for 24 h to establish infection. Following infection, the mice were randomly assigned to four treatment groups: NS, PT, PTM, and PTPM. Wound photographs were obtained on day 0, 3, 7, 10, 12, and 14 to monitor healing progression. The PTPM hydrogel significantly accelerated wound closure compared to the other treatments (Fig. 5b).

Quantitative assessment of wound closure and corresponding schematic representations further confirmed that PTPM demonstrated the most pronounced wound-healing efficacy (Fig. 5c, d). By day 7, the PTPM group showed a markedly smaller residual wound area (46.7%) compared with the PTM (66.5%), PT (80.1%), and NS (92.4%) groups, accompanied by well-formed scabs and minimal exudation. By day 14, wounds treated with PTPM were almost fully closed (healing rate > 99%), whereas the other groups exhibited delayed or incomplete healing.

To quantify in vivo antibacterial activity, wound exudates were collected on day 3 for bacterial culture (Fig. 5e). Consistent with in vitro findings, bacterial survival in the PTPM group decreased sharply to 0.38%, whereas substantially higher bacterial burdens persisted in the other groups (Fig. 5f).

Histological analyses were conducted to assess inflammation and tissue regeneration during wound repair. On day 7, hematoxylin and eosin (H&E) staining exhibited that the PTPM group exhibited the most advanced re-epithelialization, with continuous epithelial bridging and minimal necrotic or scab tissue at the wound center. Inflammatory cell infiltration and edema were significantly reduced, and granulation tissue appeared denser and more evenly distributed, indicating a well-established transition from the inflammatory to the proliferative phase. Newly formed microvessels and fibroblast clusters were evident, providing structural support for subsequent extracellular matrix (ECM) remodeling (Fig. 5g).

Masson’s trichrome staining on day 7 demonstrated significantly increased collagen deposition in the PTPM group, with continuous and aligned collagen fibers, whereas other groups showed sparse, fragmented, or irregular staining patterns (Fig. 5h). Together with the H&E results, our findings imply that PTPM hydrogel promoted earlier and more robust ECM synthesis and remodeling, accelerating wound contraction and enhancing tissue structural integrity.

By day 14, H&E staining revealed nearly complete re-epithelialization in the PTPM group, with a well-organized epidermal layer resembling intact skin. Inflammatory infiltrates were minimal, granulation tissue was mature, and the dermis exhibited abundant neovascularization with orderly fibroblast distribution (Fig. 5i). These observations aligned with immunofluorescence findings, including increased keratin 14 (K14) and CD31 expression and reduced pro-inflammatory cytokine levels, confirming superior epidermal regeneration, anti-inflammatory activity, and angiogenic capacity in the PTPM-treated group.

Masson’s trichrome staining on day 14 further demonstrated dense, continuous collagen deposition in the PTPM group, with thicker and more parallel collagen bundles indicative of advanced ECM maturation. In contrast, other groups exhibited sparse, disorganized, or fragmented collagen structures (Fig. 5j). Together, these histological findings confirm that the PTPM hydrogel effectively suppressed inflammation, enhanced re-epithelialization, and promoted collagen remodeling during diabetic wound healing.

Here, the PT group (PVA-TSPBA without MoS2/PGS) was included as a hydrogel-matrix control to account for the general benefits of hydrogel dressings (e.g., maintaining a moist environment and providing physical protection).

It should be noted that hydrogels themselves can facilitate wound healing by serving as a moist and protective dressing. This baseline dressing effect is reflected by the moderate improvement of the PT hydrogel compared with NS. Importantly, because PT, PTM, and PTPM share the same PVA-TSPBA matrix, the further enhancement observed in PTM and especially PTPM over PT cannot be explained by the presence of a hydrogel dressing alone, but rather suggests additional contributions from the incorporated nanocomposites (MoS2 and PGS/MoS2) to infection control and microenvironment regulation.

**Fig. 6:**
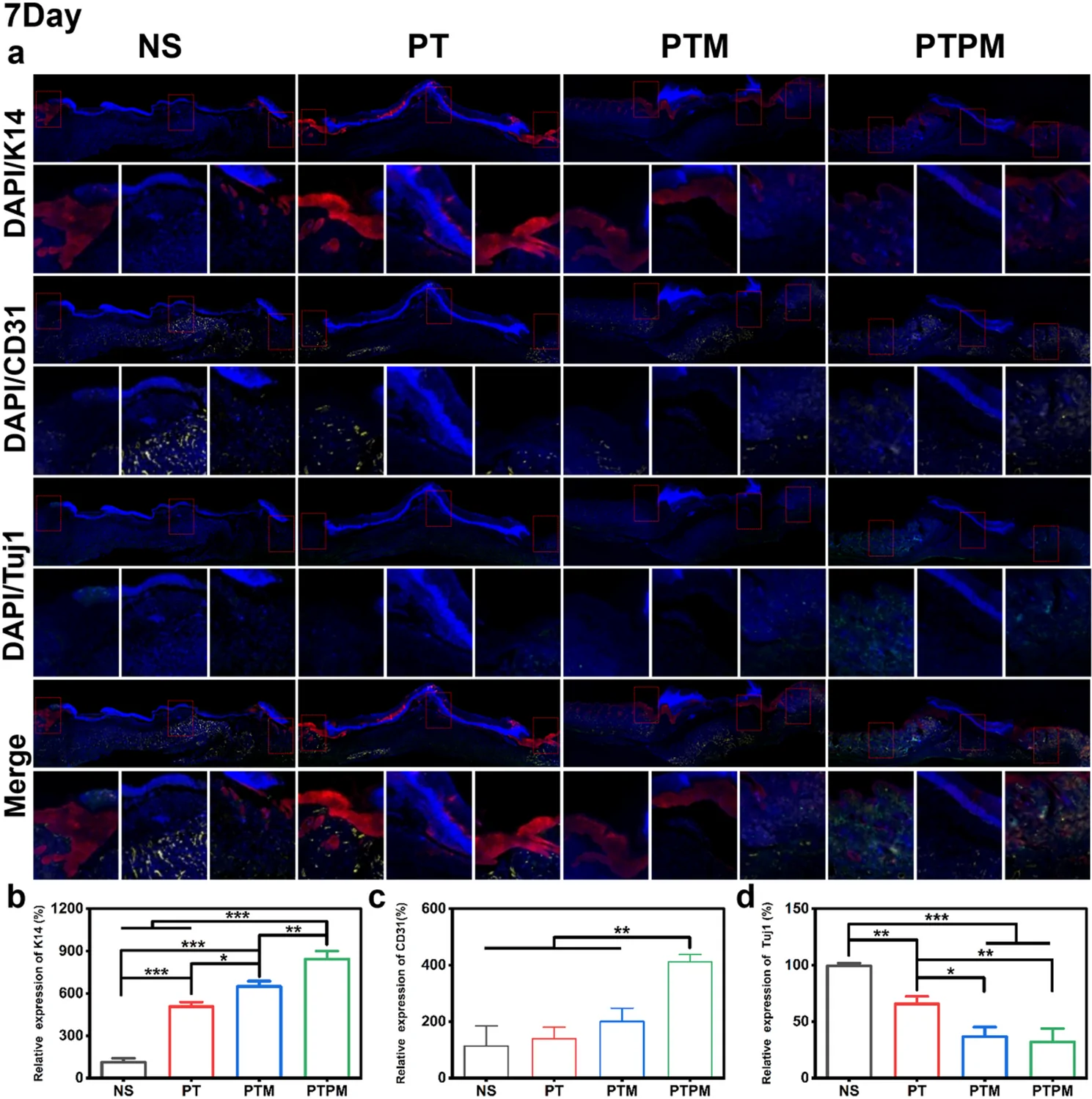
PTPM hydrogel promotes wound healing in *Staphylococcus aureus*–infected diabetic mice (Day 7). **a**) Representative immunofluorescence images of wound tissues from different treatment groups on day 7. Red fluorescence indicates K14 (epithelial marker), yellow indicates CD31 (endothelial marker), green indicates Tuj1 (neuronal marker), and blue represents DAPI-stained nuclei. The PTPM hydrogel group exhibited the most pronounced enhancement of re-epithelialization and neovascularization. **b-d**) Quantitative analysis of fluorescence areas for K14, CD31, and Tuj1 in wound tissues from each group on day 7, confirming that the PTPM hydrogel group demonstrated the strongest epithelial and vascular regeneration. (*n* = 3 per group).All data are presented as mean ± SD; *P < 0.05, **P < 0.01, ***P < 0.001.*S. aureus*, *Staphylococcus aureus*; K14, keratin-14; DAPI, 4′,6-diamidino-2-phenylindole

On day 7, immunofluorescence staining showed that the PTPM hydrogel group exhibited the strongest signals for K14, an epidermal marker, and CD31, an angiogenesis-associated marker, reflecting enhanced epidermal regeneration and neovascularization. In contrast, the normal saline (NS) group demonstrated the highest fluorescence intensity of the neuronal marker βIII-tubulin (Tuj1) (Fig. 6a). Quantitative analysis further confirmed that the PTPM group had the largest fluorescence area and highest expression levels of K14 and CD31, supporting its pronounced efficacy in promoting epithelial repair and vascular remodeling (Fig. 6b–d).

**Fig. 7:**
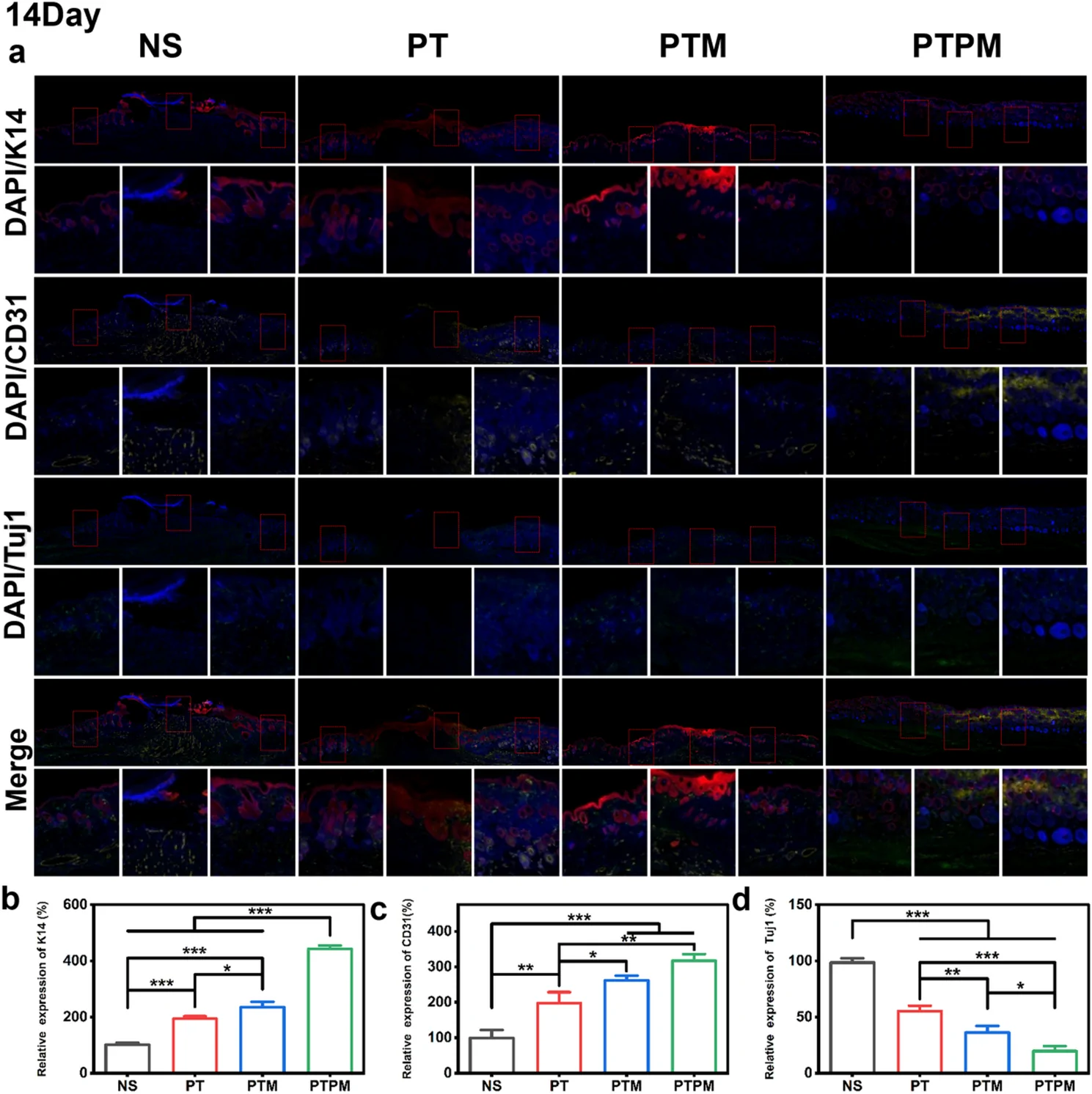
PTPM hydrogel promotes wound healing in diabetic mice with infected wounds (Day 14). **a**) Representative immunofluorescence images of wound tissues from different treatment groups on day 14. Red fluorescence indicates K14 (epithelial marker), yellow indicates CD31 (endothelial marker), green indicates Tuj1 (neuronal marker), and blue represents DAPI-stained nuclei. The PTPM hydrogel group showed the most pronounced enhancement of epidermal regeneration and angiogenesis. **b-d**) Quantitative analysis of fluorescence areas for K14, CD31, and Tuj1 in wound tissues from different treatment groups on day 14. The PTPM hydrogel group exhibited the most significant promotion of epidermal regeneration and vascular formation. (*n* = 3 per group).All data are presented as mean ± SD; *P < 0.05, **P < 0.01, ***P < 0.001

K14, keratin-14; DAPI, 4′,6-diamidino-2-phenylindole; CD31, cluster of differentiation 31.

On day 14, immunofluorescence staining demonstrated that the PTPM hydrogel group exhibited the strongest signals for K14 and CD31, reflecting the most substantial enhancement of epidermal regeneration and angiogenesis. In contrast, the NS group exhibited the highest fluorescence intensity for Tuj1 (Fig. 7a). Quantitative fluorescence area analysis further confirmed that the PTPM hydrogel group achieved the most effective restoration of epidermal and vascular structures (Fig. 7b–d).

Although the NS group displayed stronger Tuj1 fluorescence on days 7 and 14, this likely reflected inflammation-driven nerve sprouting rather than mature, functional reinnervation. Under conditions in which infection and oxidative stress were insufficiently controlled, local upregulation of neurotrophic and vasoactive mediators, such as nerve growth factor (NGF) and vasoactive intestinal peptide (VIP), may have triggered aberrant peripheral nerve proliferation and sensitization. Consequently, increased Tuj1 signals were observed; however, this response was accompanied by delayed re-epithelialization, inadequate vascular remodeling, and persistent inflammation, indicating impaired rather than effective tissue repair [30].

In contrast, treatment with the PTPM hydrogel significantly reduced early pro-inflammatory signaling through ROS scavenging, synergistic photothermal and photodynamic antibacterial activity, and immune modulation. This created a more favorable microenvironment that supported timely epidermal closure and vascular reconstruction, promoting a temporally coordinated and well-regulated pattern of reinnervation, as reflected by a non-aberrant Tuj1 distribution. These findings align with our three-dimensional (3D) tissue-clearing findings, which demonstrated denser neural branching but impaired epithelial repair in the NS group, and with molecular analyses (qPCR and Western blot) showing elevated NGF and VIP expression in the NS group alongside reduced pro-inflammatory markers and enhanced vascular and epithelial markers in the PTPM group. Overall, the lower Tuj1 fluorescence in the PTPM group reflected a more balanced progression of inflammation resolution, tissue regeneration, and remodeling, resulting in faster wound contraction, more complete re-epithelialization, and more mature, organized collagen and vascular architecture [31].

On day 14, histopathological examination of major organs, including the heart, liver, spleen, lung, and kidney, demonstrated normal tissue architecture across all groups, with no evidence of inflammatory infiltration or pathological alterations. These findings indicate the excellent biocompatibility and biosafety of the applied materials (Figure S19).

### Tissue-clearing analysis of wound repair mediated by the PTPM hydrogel

**Fig. 8:**
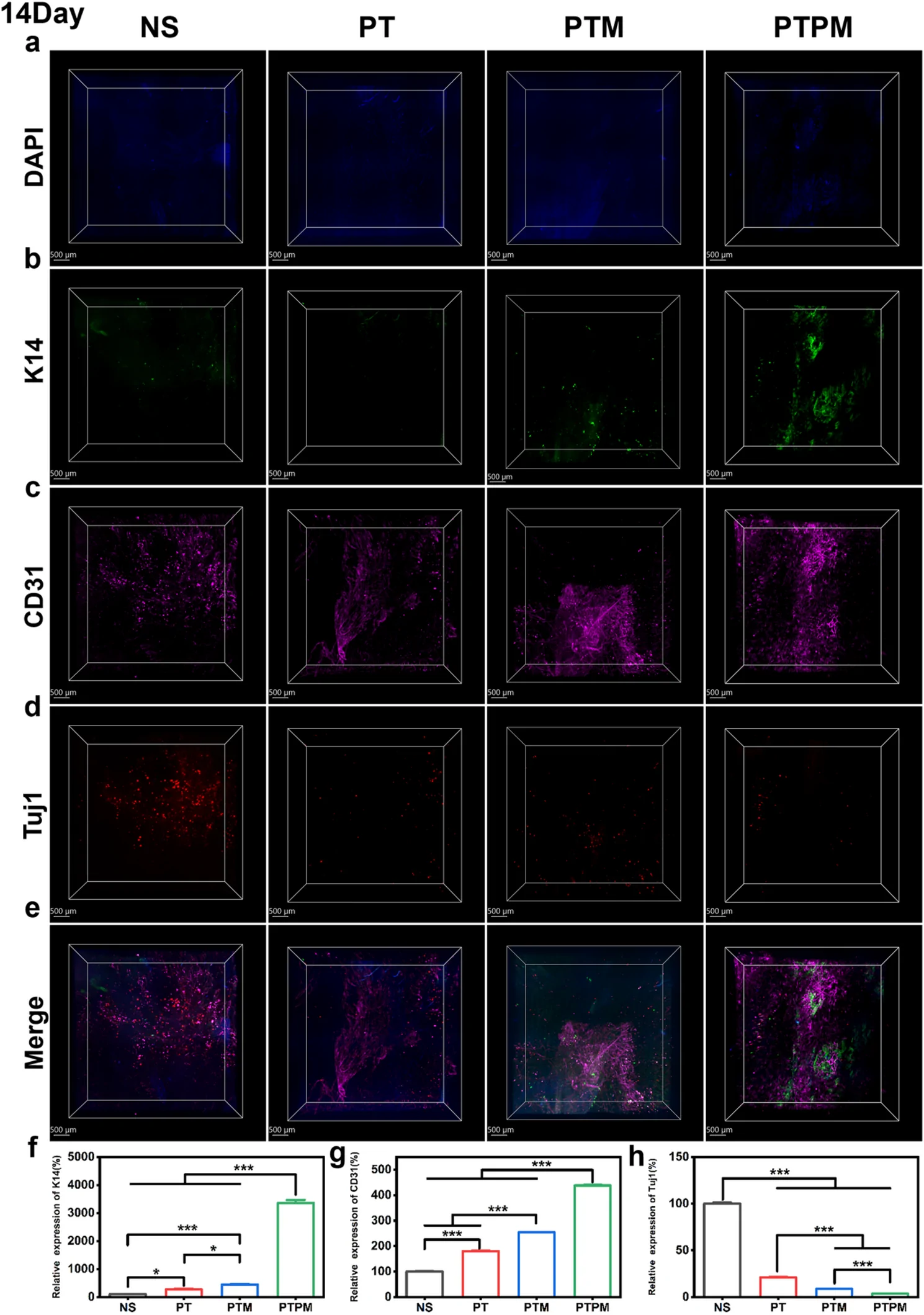
Tissue clearing reveals three-dimensional epidermal, vascular, and neural networks within wound sites. **a-e**) Representative tissue-clearing images of diabetic mouse wounds at day 14 post-treatment, showing epidermal (K14, green), vascular (CD31, violet), and neural (Tuj1, red) markers. Three-dimensional reconstructions were generated using Imaris software based on light-sheet microscopy data. Scale bar: 500 μm. The PTPM hydrogel group exhibited the most complete epidermal regeneration and the most extensive vascular network formation. **f-h**) Quantitative analysis of fluorescence areas for K14, CD31, and Tuj1 signals in cleared wound tissues on day 14 across different treatment groups. The PTPM hydrogel group exhibited significantly enhanced epidermal regeneration and angiogenesis compared to other groups. (*n* = 3 per group).All data are presented as mean ± SD; *P < 0.05, **P < 0.01, ***P < 0.001.K14, keratin-14; CD31, cluster of differentiation 31

To evaluate further the therapeutic effects of the PTPM hydrogel on infected wounds, skin tissues were harvested on day 14 post-treatment and subjected to tissue clearing for 3D imaging. The reconstructed 3D images revealed distinct structural variations in epidermal, vascular, and neural architecture among the treatment groups. Notably, the PTPM group demonstrated the most complete and continuous epidermal regeneration, whereas the NS group exhibited incomplete epithelial closure with evident defects (Fig. 8a and b and in Supplementary Videos).

3D reconstruction of the wound vasculature revealed that the PTPM group had the highest vascular branch density and greatest total vessel length, reflecting robust neovascularization (Fig. 8c and in Supplementary Videos). Conversely, neural network visualization indicated excessive and disorganized neural sprouting in the NS group, whereas the PTPM group displayed spatially confined and structurally organized nerve regrowth (Fig. 8d and e and in Supplementary Videos).

Quantitative fluorescence analysis further substantiated these findings: expression levels of K14 and CD31, markers of epidermal regeneration and vascular endothelial activity respectively, were highest in the PTPM group, whereas the neuronal marker Tuj1 was expressed at lower levels (Fig. 8f–h). This pattern indicates that the PTPM hydrogel optimally promoted epidermal repair and vascular reconstruction and supported neural regeneration in a temporally coordinated and spatially regulated manner, contributing to a balanced and functionally mature wound-healing response.

During wound repair, nerve density is typically elevated during the early inflammatory phase and subsequently declines during the remodeling phase, a trajectory influenced by inflammatory signaling, neurotrophic regulation, and matrix remodeling.

Following injury, neural remodeling exhibits a biphasic pattern. In the early phase of neural hyperplasia, inflammatory stimuli induce the release of prostaglandins [32], bradykinin [33], and neurotrophic factors, such as NGF [34] from damaged tissues and immune cells. These mediators sensitize peripheral nerve endings and promote axonal sprouting toward the wound, resulting in a transient increase in local nerve fiber density [34]. As healing advances into scar formation and tissue remodeling, a late-phase reduction in neural fibers becomes evident. The maturing fibrotic matrix replaces damaged tissue, and its dense, rigid structure can compress nearby nerve fibers, limit vascular perfusion and nutrient delivery, and ultimately promote axonal degeneration [35, 36]. In addition, the fibrotic environment restricts progenitor cell migration and axonal extension, preventing complete neural restoration and contributing to progressive decline in nerve fiber density during later stages of repair [37].

### qPCR and Western blot analysis of wound healing mediated by PTPM hydrogels

**Fig. 9:**
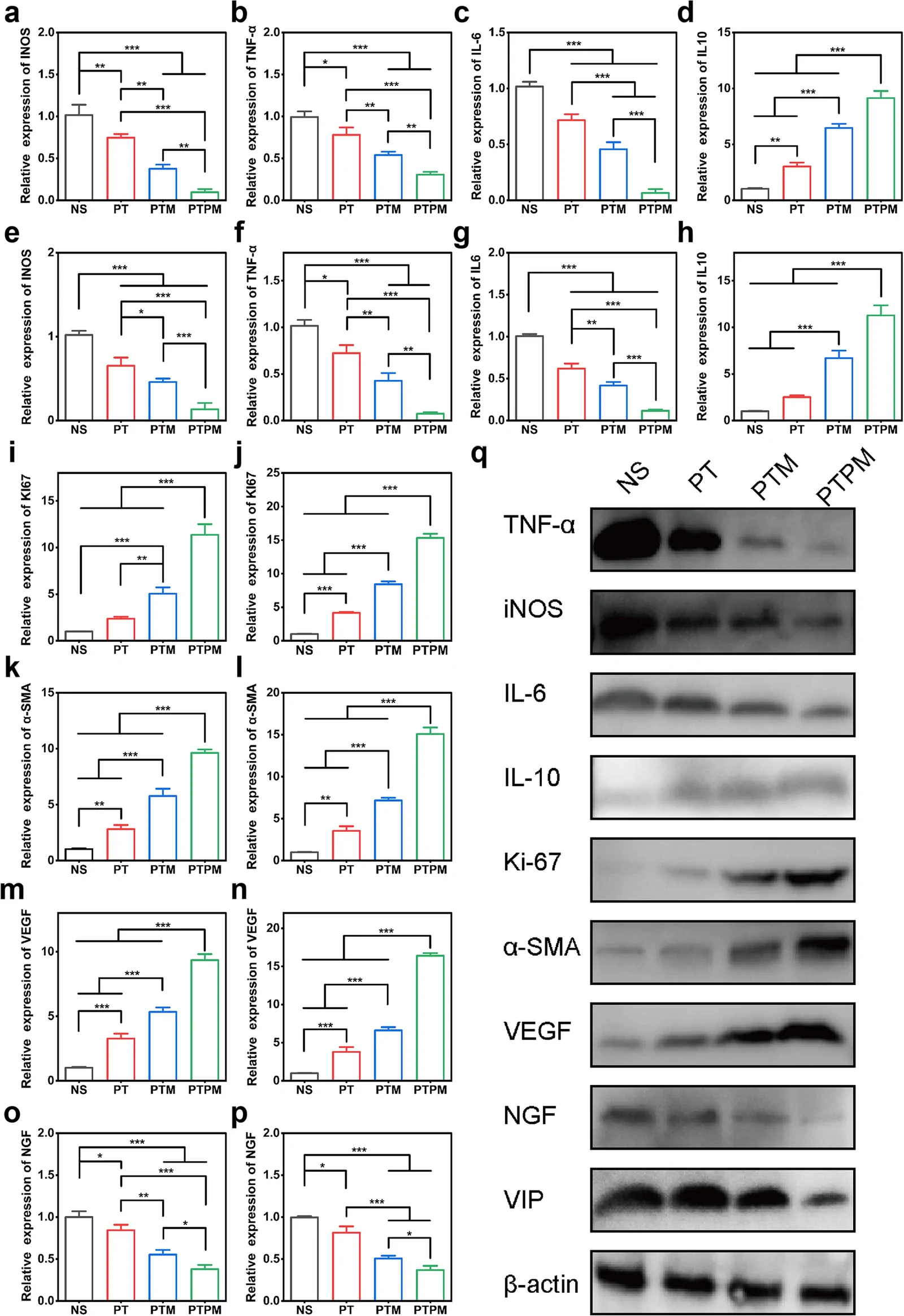
qPCR and Western blot analyses of wound healing mediated by PTPM hydrogels. **a-d**) qPCR analysis of pro-inflammatory (iNOS, TNF-α, IL-6) and anti-inflammatory (IL-10) markers in wound tissues from the different treatment groups on day 7. **e-h**) qPCR analysis of pro-inflammatory (iNOS, TNF-α, IL-6) and anti-inflammatory (IL-10) markers in wound tissues from the different treatment groups on day 14. **i-l**) qPCR analysis of epithelialization-related gene (Ki67), angiogenesis-related genes (α-SMA and VEGF), and neurogenesis-related gene (NGF) in wound tissues from different treatment groups on day 7. **m-p**) qPCR analysis of epithelialization-related gene (Ki67), angiogenesis-related genes (α-SMA and VEGF), and neurogenesis-related gene (NGF) in wound tissues from different treatment groups on day 14. All data are presented as mean ± SD. *P < 0.05, **P < 0.01, ***P < 0.001. (*n* = 3 per group) **q**) Western blot analysis of wound tissues collected on day 14, exhibiting expression levels of inflammatory and anti-inflammatory proteins (TNF-α, iNOS, IL-6, IL-10), epithelialization-related protein (KI67), angiogenesis-related proteins (NGF, VIP), and neurogenesis-related proteins under different treatments.qPCR, quantitative polymerase chain reaction; α-SMA, α-smooth muscle actin; VEGF, vascular endothelial growth factor; NGF, nerve growth factor

Chronic infectious wounds are characterized by persistent inflammation, which substantially delays tissue repair [38–40]. Bacterial colonization further exacerbates inflammation by inducing excessive production of proinflammatory cytokines [41–43]. qPCR analysis demonstrated that on days 7 and 14 post-treatment, the PTPM hydrogel group had the lowest expression of proinflammatory markers (IL-6, TNF-α, and iNOS) and the highest expression of the anti-inflammatory cytokine interleukin (IL)-10 (Fig. 9a–h). These findings indicate that PTPM hydrogels effectively suppressed inflammatory signaling, enhanced anti-inflammatory activity, and restored redox balance, facilitating wound closure through enhanced anti-inflammatory cytokine secretion [44, 45]. Furthermore, mRNA expression of angiogenesis-related genes (α-SMA and VEGF) and epithelialization markers (KI67) was significantly increased in the PTPM group at both time points, whereas expression of the neurotrophic factor NGF was reduced (Fig. 9i–p). This pattern implies that PTPM hydrogels strongly promoted angiogenesis and epithelial regeneration, with neural remodeling likely occurring at a later phase, consistent with in vitro observations.

Western blot analysis corroborated these findings; on day 14, the PTPM group exhibited the lowest protein levels of tumor necrosis factor-alpha, inducible nitric oxide synthase, and IL-6, and the highest expression of IL-10, Ki67, alpha-smooth muscle actin, and vascular endothelial growth factor (VEGF) (Fig. 9q and Figure S20). Collectively, these findings demonstrate that the PTPM hydrogels effectively attenuated inflammation and promoted vascular and epithelial regeneration, accelerating healing in infected diabetic wounds.

### RNA sequencing revealed systemic remodeling of the infectious diabetic wound microenvironment by PTPM hydrogels

**Fig. 10:**
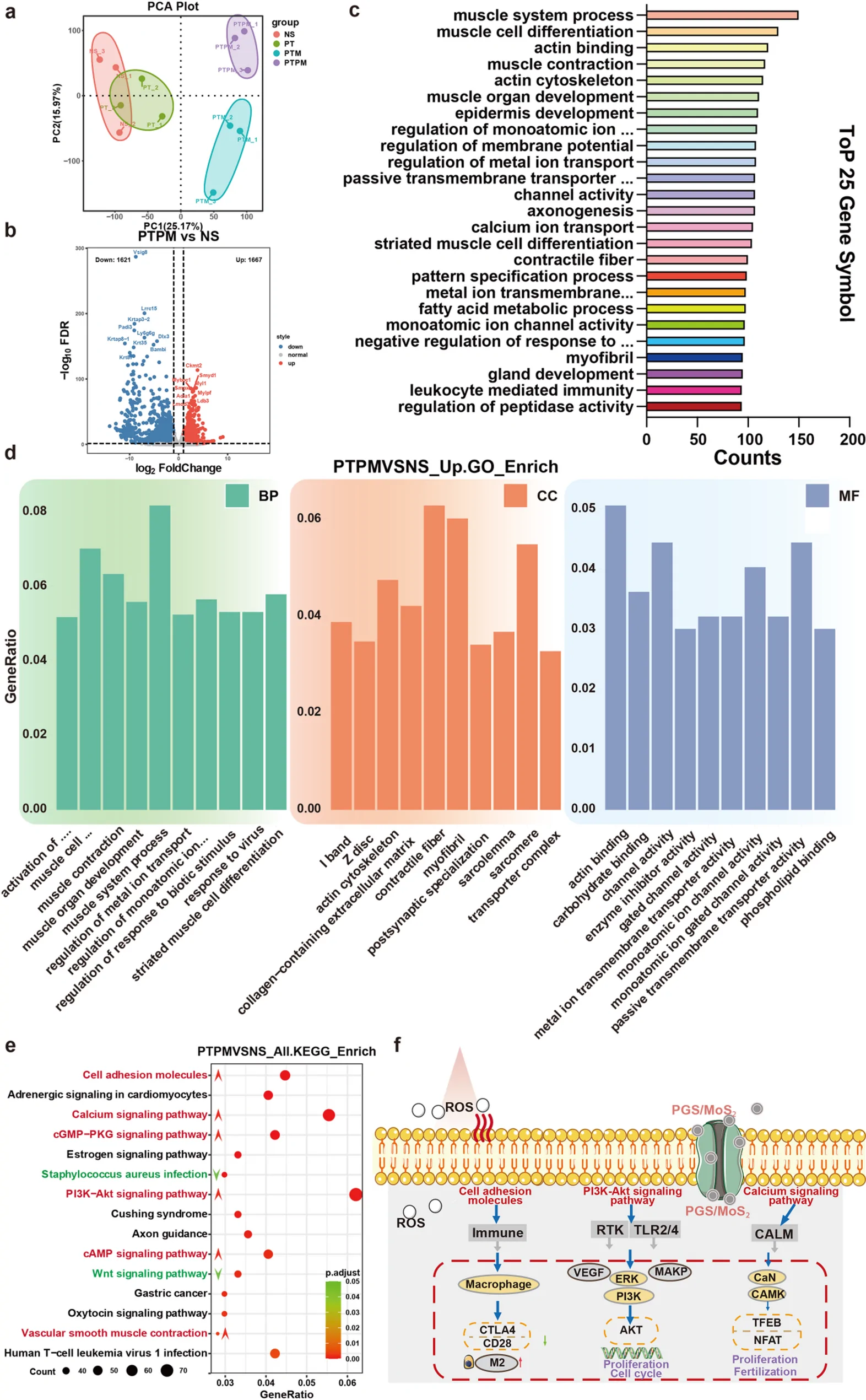
Transcriptomic analysis revealing the mechanisms by which PTPM hydrogels promote the healing of infected diabetic wounds in mice. **a**) Principal component analysis (PCA) of four groups under different treatments. The relative distance between samples reflects their similarity and potential outliers—closer proximity indicates higher similarity in gene expression profiles. **b**) Volcano plot showing differentially expressed genes (DEGs) between the PTPM and NS groups. DEGs were screened using the thresholds |log₂(Fold Change)| > 1 and *padj* < 0.05. (*n* = 3 per group). Blue dots represent downregulated genes, while red dots indicate upregulated genes. **c**) Top 25 DEGs between the PTPM and NS groups prior to hierarchical clustering. **d**) GO enrichment analysis of DEGs between the PTPM and NS groups. GO terms with *p.adjust* < 0.05 and the top 10 enriched terms by gene count are displayed, categorized into biological process (BP), cellular component (CC), and molecular function (MF). (*n* = 3 per group). **e**) KEGG pathway enrichment analysis of DEGs between the PTPM and NS groups. Pathways with *p.adjust* < 0.05 and the top 15 enriched pathways by gene count are shown. (*n* = 3 per group). Dot size represents the number of DEGs enriched in each pathway, and redder colors indicate higher significance. **f**) Schematic illustration of the potential molecular mechanism by which the PTPM hydrogel facilitates infected diabetic wound healing

We performed transcriptomic sequencing and comparative analyses of skin tissues from the four treatment groups (NS, PT, PTM, and PTPM). Principal component analysis (PCA) demonstrated a distinct separation of samples from the PTPM hydrogel group along the first principal component, indicating broad remodeling of the transcriptional landscape (Fig. 10a). Differential expression analysis using Venn diagram (Figure S21) and volcano plot outputs (Fig. 10b) revealed that PTPM treatment resulted in a substantial number of differentially expressed genes (DEGs) compared to NS. Likewise, comparisons with PT and PTM groups (Figures S22–S25) identified thousands of upregulated and downregulated genes in PTPM-treated tissues, implying a unique synergistic molecular effect derived from the combined PGS and MoS₂ components.

Hierarchical clustering further demonstrated pronounced upregulation of gene modules associated with keratinization, extracellular matrix (ECM) organization, and angiogenesis in the PTPM group, whereas genes related to inflammatory signaling and chemotaxis were broadly downregulated (Fig. 10c–d).

At the functional enrichment level, differentially expressed genes in the PTPM group were significantly enriched in key wound repair pathways, including the phosphoinositide 3-kinase (PI3K)–protein kinase B (Akt) signaling pathway, cell adhesion molecules (CAMs), and the calcium signaling pathway. Activation of the PI3K–Akt pathway drives cell proliferation, survival, and angiogenesis, consistent with the observed upregulation of Ki-67 and VEGF. Enrichment of CAMs, such as integrins and cadherins, indicates enhanced cell-cell and cell-matrix interactions, supporting keratinocyte migration, re-epithelialization, endothelial junction maturation, and vessel stabilization. In addition, activation of calcium signaling modulates cytoskeletal dynamics and cellular contractility, providing mechanical support for keratinocyte migration and wound contraction. Together, these pathways coordinate epithelialization, ECM remodeling, and angiogenesis (Fig. 10e and S23–S27).

These transcriptomic alterations align with histological, immunofluorescence, qPCR, and Western blot findings, in which inflammatory markers were suppressed and regenerative markers, including IL-10, Ki-67, alpha-smooth muscle actin, and VEGF, were substantially upregulated (Figs. 8 and 9). Mechanistically, these findings support a model in which PTPM hydrogels accelerate wound healing through complementary actions, including ROS scavenging, photodynamic and photothermal antibacterial effects, and coordinated immune and ECM remodeling.

Notably, transcriptomic comparisons revealed that the PTPM hydrogel induced thousands of unique differentially expressed genes relative to PT and PTM (Figures S22–S27), with distinct gene clusters evident in Venn and heatmap analyses. These findings indicate that incorporation of PGS and MoS₂ generates a synergistic rather than additive effect, driving systemic reorganization of regenerative signaling networks, including ECM-receptor interaction, focal adhesion, PI3K–Akt signaling, CAM pathways, and calcium signaling (Fig. 10f). Enrichment of CAM and ECM-related genes implies that PTPM accelerates epithelial closure and improves the quality and stability of re-epithelialization by reinforcing intercellular and cell-matrix adhesion. Concurrent activation of PI3K–Akt and calcium signaling, key regulators of cellular migration, proliferation, and differentiation, provides strong molecular impetus for tissue regeneration. These transcriptional programs are consistent with the hydrogel’s advantageous physicochemical features, including photothermal and photodynamic responsiveness and favorable adhesive and rheological behavior (Fig. 2).

Collectively, RNA sequencing analysis demonstrated that the PTPM hydrogel orchestrated coordinated activation of PI3K–Akt, CAM, and calcium signaling pathways while suppressing inflammation, coupling resolution of inflammation with enhanced epithelialization, ECM remodeling, and angiogenesis. This integrated “inflammation-resolution and regeneration-promotion” effect establishes a pro-healing molecular microenvironment and mechanistically explains the superior in vivo therapeutic efficacy of PTPM.

In this study, we compared the therapeutic effects of four hydrogel treatments—Normal Saline (NS), PVA-TSPBA (PT), PVA-TSPBA@MoS₂ (PTM), and PVA-TSPBA@PGS/MoS₂ (PTPM)—on diabetic wound healing. The data revealed significant differences between the groups across multiple parameters(Table 1).

**Table 1:** Comparison of PTPM Hydrogel's Treatment Effects with NS, PT, and PTM Groups

Treatment Group	NS (Normal Saline)	PT (PVA-TSPBA )	PTM (PVA-TSPBA@ MoS₂)	PTPM (PVA-TSPBA@ PGS/MoS₂ )
Treatment Group	NS(Normal Saline)	PT (PVA-TSPBA )	PTM (PVA-TSPBA@ MoS₂)	PTPM (PVA-TSPBA@PGS/MoS₂ )
Gelation Time(min)*	-	33.8 ± 1.4	24.5± 1.2	19.4 ± 1.0
Photothermal Peak Temp (°C)	27.62±0.27	29.14±0.13	38.21±0.31	47.90±0.26
ROS# Scavenging(%)	0.37 ± 0.47	19.66 ± 7.71	31.19 ± 3.09	38.93 ± 3.05
LogCFU Remaining(NIR+)	6.1450 ± 0.0054	5.6145 ± 0.0287	4.9713 ± 0.0595	3.4355 ± 0.1970
LogCFU Remaining(NIR-)	6.1739 ± 0.0018	5.7253 ± 0.0295	5.1203 ± 0.0401	4.1423 ± 0.0571
Wound Area Day 7 (%)	92.4±7.1	80.1±6.6	66.5±5.9	46.7±5.6
Wound Area Day 14 (%)	49.5±8.7	40.2±7.7	23.9±6.0	5.1±4.1

The rapid gelation of PTPM is particularly beneficial for quick wound coverage and protection, as well as for experimental operations* [46]; below the 50 °C safety threshold, ensuring biocompatibility for wound and in vivo studies# [28]

The rapid gelation of PTPM is particularly beneficial for quick wound coverage and protection, as well as for experimental operations* [46]; below the 50 °C safety threshold, ensuring biocompatibility for wound and in vivo studies# [28]

#### Gelation time

The PTPM hydrogel exhibited the fastest gelation time (19.4 ± 1.0 min), followed by PTM (24.5 ± 1.2 min) and PT (33.8 ± 1.4 min). NS did not form a gel. The rapid gelation of PTPM is particularly beneficial for quick wound coverage and protection, as well as for experimental operations. This result is consistent with recent studies, which report that hydrogels enhanced with nanoparticles can accelerate gelation times, benefiting experimental handling [46].

#### Photothermal response

PTPM showed the highest photothermal peak temperature (47.90 ± 0.26 °C), promoting wound healing by exerting antibacterial effects through mild photothermal reactions. This was followed by PTM (38.21 ± 0.31 °C) and PT (29.14 ± 0.13 °C), while NS showed almost no photothermal response (27.62 ± 0.27 °C). Recent studies have shown that photothermal hydrogels incorporating MoS₂ nanoparticles exert antibacterial effects through thermal action, significantly promoting the healing of diabetic wounds [47].

#### ROS scavenging efficiency

PTPM exhibited the highest ROS scavenging efficiency (38.93 ± 3.05%), followed by PTM (31.19 ± 3.09%) and PT (19.66 ± 7.71%). NS showed no ROS scavenging (0.37 ± 0.47%). The high ROS scavenging ability of PTPM suggests its particular effectiveness in managing oxidative stress in diabetic wounds, where excessive ROS production impedes healing. Previous studies have also shown that hydrogels with enhanced ROS scavenging can reduce oxidative damage, thereby promoting wound healing [48].

#### Antibacterial effectiveness

The PTPM hydrogel demonstrated the most significant antibacterial effects, with LogCFU remaining of 3.4355 ± 0.1970 under NIR + and 4.1423 ± 0.0571 under NIR- conditions. PTM and PT showed moderate antibacterial effects, while NS demonstrated minimal antibacterial activity. These findings are consistent with other research, indicating that hydrogels incorporating MoS₂ nanoparticles exhibit significant antibacterial effects under near-infrared light, greatly facilitating the treatment of infected diabetic wounds [49] .

#### Wound healing

In terms of wound area reduction, PTPM exhibited the most significant healing on Day 7 (46.7 ± 5.6%) and Day 14 (5.1 ± 4.1%). PTM and PT also showed significant healing, though to a lesser degree. NS showed the poorest healing (92.4 ± 7.1% on Day 7 and 49.5 ± 8.7% on Day 14). These results align with the application of multifunctional hydrogels in diabetic wound treatment, demonstrating that by promoting tissue regeneration, reducing bacterial load, and managing oxidative stress, wound healing can be significantly improved [50] .

Overall, the PVA-TSPBA@PGS/MoS₂ hydrogel exhibited superior performance across all evaluated parameters—rapid gelation, high photothermal response, effective ROS scavenging, strong antibacterial activity, and significant wound healing effects. These results are consistent with recent literature, further validating the effectiveness of multifunctional hydrogels in treating diabetic infections. The unique combination of properties in this hydrogel provides strong support for advancing diabetic wound care.

## Conclusion

We developed an intelligent injectable composite hydrogel (PTPM) that combines strong tissue adhesion with photodynamic and mild photothermal responsiveness. Incorporation of PGS/MoS₂ nanomaterials into the PVA-TSPBA network substantially enhanced the hydrogel’s angiogenic, antioxidant, and antibacterial capacities. In vitro assays demonstrated that PTPM effectively scavenged ROS, promoted cell migration and angiogenesis, and exhibited robust bactericidal activity against *S. aureus*, while maintaining excellent biocompatibility. In a diabetic mouse model with infected wounds, PTPM significantly accelerated wound repair by enhancing epithelialization and neovascularization, accompanied by potent antibacterial efficacy, favorable biosafety, and minimal systemic toxicity.This multifunctional hydrogel shows great potential as a therapeutic platform for treating diabetic wounds, effectively promoting wound healing. Future studies will focus on clinical validation, particularly in human trials, to assess its efficacy and safety in different diabetic wound healing scenarios. Additionally, research will explore its performance in real-world clinical settings to confirm its feasibility and stability for long-term use. These further investigations are crucial for advancing the clinical translation of this hydrogel.

Overall, this multifunctional hydrogel represents a promising therapeutic platform for targeted microenvironment modulation and accelerated repair of infected diabetic wounds, offering a potential alternative strategy for the clinical management of chronic infectious wounds.

## Materials and methods

### Materials

The murine fibroblast cell line L929 (Cat. No. CL-0137) and fetal bovine serum (FBS, Cat. No. 164210) were obtained from Wuhan Procell Life Science & Technology Co., Ltd. (Wuhan, China). Endothelial growth medium (EGM-2, Cat. Nos. CC-5156 + CC-4176) was purchased from Beijing Zeping Technology Co., Ltd. (Beijing, China). Dulbecco’s Modified Eagle Medium (DMEM, Cat. No. 0125018) was obtained from Thermo Fisher Scientific (Massachusetts, USA). Absolute ethanol (Cat. No. E809063) was purchased from Macklin Biochemical Technology Co., Ltd. (Shanghai, China). Cell Counting Kit-8 (CCK-8, Cat. No. C0037) and the Cell Viability and Cytotoxicity Assay Kit (Cat. No. C2015S) were obtained from Beyotime Biotechnology (Shanghai, China).

4-(Bromomethyl)phenylboronic acid (Cat. No. B165281), N, N-dimethylformamide (Cat. No. D112007), N, N,N′,N′-tetramethyl-1,3-propanediamine (Cat. No. T106825), PVA (Cat. No. P105126), MoS₂ (Cat. No. M431857), and tetrahydrofuran (THF, Cat. No. T120775) were purchased from Aladdin Biochemical Technology Co., Ltd. (Shanghai, China). Palygorskite (PGS) was obtained from Xuyi Aotu Co., Ltd. (Jiangsu, China). C57BL/6J mice were obtained from SPF Biotechnology Co., Ltd. (Beijing, China).

### General methods

#### Synthesis and characterization of PVA-TSPBA hydrogels with varying ratios

##### Preparation of PVA-TSPBA hydrogels

PVA-TSPBA hydrogels were synthesized using a precipitation-based method for preparation of the crosslinker TSPBA. Briefly, 2.3 mmol of 4-(bromomethyl)phenylboronic acid and 0.75 mmol of tetramethylethylenediamine were added to a 25-mL round-bottom flask containing 10 mL of N, N-dimethylformamide (DMF). The mixture was stirred and maintained at 60 °C overnight. After cooling to room temperature, 100 mL of THF was added to induce precipitation. The resulting white solid was collected via vacuum filtration through a sintered glass funnel and washed three times with 60 mL of THF. The purified product was dried to obtain TSPBA for subsequent hydrogel formation.

##### Solid-state ¹H NMR analysis

Solid-state ¹H NMR analysis was performed using a Bruker Avance Neo 400WB spectrometer (Bruker Corp., Billerica, MA, USA) equipped with an H/X double-resonance probe. Samples were placed into 4-mm zirconia rotors and gently compacted with a loading rod. Spectra were acquired at a spinning rate of 8 kHz with 32 scans per sample. Instrument settings included a resonance frequency of 400.33 MHz, a pulse width of 2.5 µs, a relaxation delay of 3 s, and an acquisition time of 0.06 s per scan. A single-pulse sequence was applied, and data processing was conducted automatically using the instrument’s acquisition software (TopSpin, Bruker Corp.,).

##### Solution-state NMR analysis

Solution-state NMR analysis was carried out using a Bruker Avance Neo 600 MHz spectrometer (Bruker Corp.,). Samples were dissolved in deuterated solvents prior to measurement. Data were collected with a spectral width of 10,000 Hz, 16 scans per sample, and a relaxation delay of 5 s. Spectral processing, including baseline correction, phase adjustment, chemical-shift referencing, and peak integration, was performed using MestReNova software (Mestrelab Research SL, Santiago de Compostela, Spain). The degree of substitution or grafting was quantified based on integrated signal intensities.

##### PVA solutions and gelation assessment

Poly(vinyl alcohol) (PVA, 30 g) was dissolved in 100 mL of distilled water at 90 °C and filtered through a 0.22-µm cellulose acetate membrane to remove insoluble residues, yielding a 30 wt% clear PVA solution. Serial dilutions (1%, 5%, 10%, 20%, and 30%, w/w) were prepared and evaluated for injectability, gelation time, and gel formation. Because the introduction of additional components shortened gelation time and reduced injectability, 10%, 15%, and 20% PVA solutions were selected for subsequent experiments.

Aqueous solutions of TSPBA (5%, 10%, and 15%, w/w) were prepared and mixed with the selected PVA solution (10%, 15%, and 20%, w/w) at a 1:3 volume ratio. Gelation behavior and gelation times were recorded to identify optimal gelation conditions.

#### Preparation of MoS₂/PGS composite materials with different ratios

MoS₂/PGS composites were synthesized via a one-step hydrothermal method. PGS powders were thermally treated (heating rate: 10 °C min⁻¹ to 300 °C, maintained for 2 h) and subsequently acid-treated in 3 mol L⁻¹ hydrochloric acid under continuous stirring for 3 h. The product was washed to neutral pH, dried at 60 °C for 8 h, and ground into a fine powder.

The PGS powder and MoS₂ were dispersed in anhydrous ethanol (Merck, Darmstadt, Germany), with a PGS mass fraction of 2.5%, 5%, 7.5%, and 10% relative to MoS₂. The suspensions were ultrasonicated for homogeneous dispersion, stirred thoroughly, and transferred into 100-mL Teflon-lined stainless-steel autoclaves (Parr Instrument Co., Moline, IL, USA). The hydrothermal reaction was conducted at 220 °C for 24 h. The resulting composites were collected by centrifugation, washed repeatedly, and freeze-dried for further use.

##### SEM

The morphology and elemental distribution of MoS₂/PGS composites with varying PGS mass ratios were examined using a TESCAN MIRA LMS SEM (TESCAN, Brno, Czech Republic). Samples were sputter-coated with gold (Quorum SC7620, 10 mA, 45 s) prior to imaging. SEM micrographs were acquired at 3 kV (secondary electron detector, SE2), and energy-dispersive X-ray spectroscopy mapping was performed at 15 kV.

##### XRD

XRD patterns of MoS₂/PGS composites with different PGS mass fractions were recorded using a Bruker D8 Advance diffractometer (Bruker Corp.) operating at 40 kV and 40 mA. Diffraction data were collected over a 2θ range of 5–90° at a scanning rate of 1° min⁻¹. The diffraction profiles were analyzed to determine phase composition and crystallinity.

#### Photothermal and photodynamic properties of MoS₂/PGS composites with varying ratios

##### Photothermal performance

The photothermal properties of MoS₂/PGS composites with varying mass ratios were evaluated under 808-nm NIR laser irradiation. Each sample was evenly spread on a paper substrate and irradiated with an 808-nm NIR laser (1.0 W cm⁻²) for 10 min. Real-time temperature evolution was recorded using an infrared thermal imaging camera to assess photothermal conversion efficiency.

##### Photodynamic performance

The generation of hydroxyl radicals (•OH) and singlet oxygen (¹O₂) under NIR irradiation was quantified by monitoring the degradation of MB and DPBF, respectively.

For ¹O₂ detection, 1 mg of each composite was added to 20-mg L⁻¹ DPBF ethanol solution (3 mL) and irradiated under identical conditions. The degradation of DPBF was measured by UV–Vis spectroscopy to determine ¹O₂ generation efficiency. The photodynamic performances of composites with different PGS/MoS₂ ratios were compared based on their relative •OH and ¹O₂ production.

For •OH detection, 1 mg of each MoS₂/PGS composite was dispersed in 5 mg L⁻¹ MB solution (3 mL) and irradiated with the 808-nm laser(1.0 W·cm⁻²) for 10 min. The supernatant was collected by centrifugation, and the remaining MB concentration was measured by UV–Vis spectroscopy to quantify •OH production.

#### Fabrication and characterization of PTPM injectable hydrogels

##### Hydrogel preparation

MoS₂/PGS composites with varying mass ratios were incorporated into the PVA-TSPBA aqueous solution. The mixture was heated at 90 °C for 15 min to induce crosslinking, resulting in the formation of PTPM injectable hydrogels. Among the tested formulations (using MB and DPBF assays), the 15 wt% PVA–5 wt% TSPBA system exhibited the most favorable gelation time. Photodynamic evaluation demonstrated that the 15 wt% PVA–5 wt% TSPBA hydrogel exhibited the highest ROS generation under NIR irradiation. The prepared hydrogel was loaded into a syringe and extruded to assess injectability, gelation behavior, and structural integrity.

##### Mechanical and rheological characterization

###### Mechanical flexibility test

The adhesion and flexibility of the PTPM hydrogel were evaluated by applying it to a human fingertip. The hydrogel maintained intimate contact with the skin during repeated bending, demonstrating excellent mechanical compliance and interfacial stability.

###### Rheological measurements

Rheological properties were measured using an MCR 302 rheometer (Anton Paar, Graz, Austria) equipped with a 50-mm parallel-plate geometry and a 1-mm gap. The system was preheated for 30 min, and all measurements were performed at 25 °C using the CP25 rotor (25 mm diameter).

###### Viscosity tests

Hydrogels were evenly spread on the lower plate, and shear viscosity was recorded under a constant shear rate of 5 s⁻¹ for 10 min. Viscosity–time profiles were plotted using the rheometer software (Anton Paar).

###### Viscoelastic modulus tests

The storage modulus (G′) and loss modulus (G″) were measured under an angular frequency of 6.28 rad s⁻¹ and a shear strain of 5% for 10 min. Temporal evolution of G′ and G″ was analyzed to assess the hydrogel’s viscoelastic behavior.

###### Strain-dependent cyclic tests

Alternating strain amplitudes of 1% and 300% were applied under an angular frequency of 6.28 rad s⁻¹ for 10 min using an MCR 302 rheometer. The corresponding variations in G′ and G″ were recorded to evaluate self-recovery and mechanical resilience.

###### FTIR spectroscopy

Freeze-dried hydrogel samples were prepared by vacuum freeze-drying and ground with anhydrous potassium bromide. The resulting powder mixtures were pressed into pellets and analyzed using a Nicolet iS20 FTIR spectrometer (Thermo Fisher Scientific, Waltham, MA, USA). Spectra were collected over 400–4,000 cm⁻¹ with a resolution of 4 cm⁻¹ and 32 scans per sample after background subtraction.

#### In vitro evaluation of PTPM hydrogels: angiogenesis assay, ROS-scavenging assay, cell migration assay, cell proliferation assay, cytotoxicity assay, and anti-inflammatory qPCR assayIn vitro evaluation of PTPM hydrogels: angiogenesis assay, ROS-scavenging assay, cell migration assay, cell proliferation assay, cytotoxicity assay, and anti-inflammatory qPCR assay

##### Angiogenesis evaluation of PTPM hydrogels

The angiogenic potential of PTPM hydrogels was assessed using a tube-formation assay with HUVECs (ATCC, Manassas, VA, USA). Matrigel (Corning, Corning, NY, USA) was thawed at 4 °C and added (80–120 µL per well) to 48-well plates on ice, followed by incubation at 37 °C for gelation. HUVECs were seeded onto the Matrigel (5 × 10⁴ cells per 200 µL well), and hydrogels with different MoS₂/PGS loadings were placed in the wells. After 24 h of incubation at 37 °C, tube formation was visualized and quantified to evaluate hydrogel-mediated pro-angiogenic activity.

##### ROS-scavenging assay

The ROS-scavenging capability of PTPM hydrogels was evaluated using an oxidative stress model in L929 fibroblasts (ATCC). L929 cells were seeded into six-well plates at a density of 2 × 10⁵ cells per well and incubated for 24 h to allow adhesion. Hydrogen peroxide (H₂O₂) was added to achieve a final concentration of 200 µmol L⁻¹ to induce oxidative stress. Next, hydrogels were introduced into the wells and co-incubated for 3 h.

Positive and negative control groups were treated similarly, except that no hydrogen peroxide was added to the negative control and no hydrogel was added to the positive control. After incubation, the culture medium was removed, and cells were stained using 20 µmol L⁻¹ DCFH-DA probe (Beyotime) for ROS detection. Intracellular fluorescence was observed and imaged using a fluorescence microscope to assess ROS-scavenging efficiency.

##### Cell migration assay

The pro-migratory effect of PTPM hydrogels was investigated using a scratch wound-healing assay. Conditioned medium was prepared by incubating hydrogels in standard L929 culture medium for 24 h. L929 cells were seeded into six-well plates (2 × 10⁵ cells per well) and cultured until a confluent monolayer formed. A uniform scratch was created using a sterile pipette tip, and wells were gently rinsed with phosphate-buffered saline (PBS) to remove detached cells. Conditioned medium was added, and cell migration was recorded at 0 h and 24 h using an inverted optical microscope (Nikon Corporation, Tokyo, Japan). Migration rates were quantified by measuring scratch closure areas using ImageJ software (National Institutes of Health, Bethesda, MD, USA).

##### Cell proliferation and cytotoxicity assay

The cell proliferation and cytotoxicity of L929 fibroblasts exposed to PTPM hydrogels were assessed using the CCK-8 assay. Conditioned medium was prepared as described above. L929 cells (5 × 10³ cells per well, 100 µL per well) were seeded into 96-well plates and cultured with hydrogel-conditioned medium. At 1, 3, and 5 day, 10 µL of CCK-8 reagent was added to each well and incubated at 37 °C for 2 h. Absorbance was measured at 450 nm using a microplate reader (Bio-Rad Laboratories, Hercules, CA, USA). Cell viability was calculated relative to the untreated control group, and the time point showing the most significant differences was selected for further analysis.

##### qPCR analysis

qPCR was performed to evaluate cellular gene expression responses after hydrogel exposure. Cells were cultured and treated as described for the CCK-8 assay. Following treatment, wells were washed three times with PBS, and 1 mL of TRIzol reagent (Invitrogen, Carlsbad, CA, USA) was added to lyse the cells. The lysate was transferred to 1.5-mL microcentrifuge tubes, mixed with chloroform, and incubated on ice for 3 min. Samples were centrifuged at 12,000 × g for 10 min at 4 °C, after which the aqueous phase was collected, mixed with isopropanol, and incubated on ice for 10 min to precipitate RNA.

The RNA pellet was washed twice with anhydrous ethanol, centrifuged, air-dried, and redissolved in RNase-free water.

The RNA pellet was washed twice with anhydrous ethanol, centrifuged, air-dried, and dissolved in RNase-free water. RNA concentration and purity were quantified spectrophotometrically. Complementary DNA was synthesized using a PrimeScript RT reagent kit (Takara Bio, Shiga, Japan). qPCR was performed using the QuantStudio 3 Real-Time PCR System (Thermo Fisher Scientific) to determine target gene expression levels. Relative expression was calculated after normalization to housekeeping genes.

#### Photodynamic antibacterial performance of PTPM hydrogels

The photodynamic antibacterial activity of PTPM hydrogels was evaluated against *Staphylococcus aureus (S. aureus)*. Bacterial suspensions (1.5 × 10^6^ CFUs) were mixed with 150 µL of hydrogel samples; the NIR(+) group was exposed to NIR irradiation (808 nm,1.0 W·cm⁻², 10 min), while the NIR(˗) group was maintained in the dark under identical conditions. After treatment, 15 µL of each bacterial suspension was spread onto LB agar plates and incubated at 37 °C for 18 h. Colony-forming units were counted to determine bactericidal efficiency.

##### SEM analysis

For SEM, fresh samples (approximately 1 mm³ of tissue or approximately 10⁷ bacteria, with pellet size not exceeding that of a soybean) were immediately fixed in 2.5% glutaraldehyde at 4 °C for 12–24 h. Fixed samples were washed three times (15 min each) with 0.1 M PBS and dehydrated in a graded ethanol series (30%, 50%, 70%, 80%, 90%, 95%, and 100%) (15 min each), followed by two 20-min washes in 100% ethanol. Samples were dried using a critical-point dryer (Quorum K850), mounted on aluminum stubs with conductive carbon tape, and sputter-coated with platinum using a sputter coater (Hitachi MC1000, ~ 120 s). Morphologies were examined using a field-emission SEM (Hitachi SU8100, Oxford UltimMax65 EDS).

##### Transmission electron microscopy analysis

For transmission electron microscopy, fresh samples (~ 1 mm³, containing ~ 10⁷ *S. aureus*) were immediately fixed in 2.5% glutaraldehyde at 4 °C for 24 h and dehydrated sequentially in 30%, 50%, 70%, and 90% ethanol (15 min each), followed by two 20-min washes in 100% ethanol and two 20-min washes in 100% acetone. Samples were infiltrated and embedded in resin through the following protocol: acetone: resin 1:1 (37 °C, 3 h); acetone: resin 1:3 (37 °C, 4 h); and pure resin overnight at 37 °C, followed by polymerization at 70 °C for 12–48 h. Ultrathin Sects.  (70–90 nm) were obtained using an ultramicrotome (Leica Microsystems), placed on copper grids, and stained with uranyl acetate (8–15 min) and lead citrate (5–10 min). After air-drying, sections were imaged using a TEM (Hitachi HT7800, 80 kV).

##### Live/dead bacterial viability assay via confocal laser scanning microscopy (CLSM)

Bacteria treated with hydrogels were cultured to the logarithmic growth phase, centrifuged, and washed two to three times with PBS. The bacterial pellets were stained with a mixture of SYTO 9 and propidium iodide (PI) working solutions and incubated in the dark at room temperature for 15–30 min with gentle vortexing. After staining, 10 µL of each suspension was placed on a glass slide, covered with a coverslip, and examined using a confocal laser scanning microscope (Leica Microsystems). Green fluorescence(live bacteria) was excited at 488 nm (SYTO 9), and red fluorescence (dead bacteria) was excited at 535 nm (PI). Images were captured after adjusting imaging parameters for optimal resolution.

#### In vivo evaluation of PTPM hydrogel for diabetic wound healing

C57BL/6 diabetic mice were used for in vivo wound-healing studies. The diabetes mouse model was established using a combination of diet and streptozotocin (STZ). The specific steps are as follows: STZ was dissolved in a citrate buffer (pH = 4) to prepare an injection solution with a concentration of 10 mg/mL. The solution was filtered through a 0.22 μm filter to remove any bacterial contaminants. After a 12-hour fasting period, the mice were injected with the prepared STZ solution at a dosage of 120 mg/kg. Fasting blood glucose levels were measured on the 3rd and 7th days after injection.

Mice with fasting blood glucose levels exceeding 16.7 mmol L⁻¹ were considered diabetic. Full-thickness excisional wounds (6 mm in diameter) were created on the dorsal skin using a biopsy punch. To establish an infectious diabetic wound model, 15 µL of *S. aureus* suspension (1 × 10⁸ CFU/mL) was applied to each wound surface and incubated for 24 h.

##### Evaluation of wound healing in vivo

The infected diabetic mice were randomly assigned to four treatment groups (*n* = 10 per group) and treated as follows:

Group 1: Normal saline (NS)

Group 2: PVA-TSPBA hydrogel (PT)

Group 3: PVA-TSPBA@MoS₂ hydrogel (PTM)

Group 4: PVA-TSPBA@PGS/MoS₂ hydrogel (PTPM)

150 µL of hydrogel formulation was directly injected onto the wound surface using a syringe, where in situ gelation occurred. Each time, the hydrogel was in contact with the wound for 2 days. The hydrogel injection was performed under anesthesia, after which the mice were allowed to wake up and move around freely with the hydrogel on their back. Additionally, 808 nm NIR light was used to activate the photothermal and photodynamic effects on the hydrogel (or normal saline), with a power density of 1.0 W·cm⁻² for 10 min.

###### Wound healing assessment

Wound areas were documented at predetermined time points, and the healing rate was calculated using the following equation:

Healing rate (%) = [(Initial wound area − Wound area on day *n*) / Initial wound area] × 100%.

On day 3 post-treatment, wound exudates were collected, serially diluted, and plated onto LB agar. After incubation at 37 °C for 18 h, bacterial colonies were counted to determine antibacterial efficacy.

On days 7 and 14, wound tissues were harvested for histological examination. H&E and Masson’s trichrome staining were performed to assess re-epithelialization and collagen deposition, respectively.

###### Epidermal regeneration, angiogenesis, and neurogenesis

At days 7 and 14, wound tissues were collected for immunofluorescence analysis. Sections were stained with primary antibodies against keratin-14 (K14, epidermal regeneration marker), CD31 (endothelial cell marker), and Tuj1 (neuronal marker). Fluorescence signal intensities were quantified to compare epidermal repair, neovascularization, and nerve regeneration across treatment groups.

##### Mechanistic investigation of PTPM-mediated wound healing

###### Western blot and reverse transcription (RT)-qPCR analyses

Wound tissues were harvested and stored at ˗ 80 °C for molecular analysis. Samples were lysed in 400 µL of lysis buffer (supplemented with PMSF, 10 µL/mL) on ice for 30 min, homogenized, and sonicated twice (1 min each). Lysates were centrifuged(12,000 rpm, 15 min, 4 °C), and the resulting supernatants were collected for downstream assays.

Western blotting and RT-qPCR were performed to quantify gene and protein expression associated with inflammation (iNOS, TNF-α, IL-6, IL-10), angiogenesis(α-SMA, VEGF), neurogenesis (NGF, VIP), and epidermal proliferation (Ki67). These analyses enabled a comprehensive evaluation of the hydrogel’s wound-healing efficacy and its regulatory effects on the local tissue microenvironment.

###### RNA sequencing and bioinformatics analysis

On day 14 post-treatment, wound tissues were collected for transcriptomic analysis. Total RNA was extracted using TRIzol™ reagent (Thermo, 15596018) according to the manufacturer’s protocol. RNA concentration and integrity were assessed using NanoDrop spectrophotometry and agarose gel electrophoresis.

Messenger RNA was enriched using poly(A) selection and used for library construction, followed by paired-end sequencing on the DNBSEQ-T7RS platform (BGI, Shenzhen, China). Raw reads were filtered to remove adapter sequences and low-quality bases. Clean reads were aligned to the mouse reference genome (mm10) using HISAT2, and gene-level quantification was performed using featureCounts. Differential gene expression was analyzed in DESeq2 (R software; R Foundation for Statistical Computing, Vienna, Austria), using |log₂ fold change| > 1 and adjusted *p* < 0.05 as the significance criteria. (*n* = 3 per group).

Volcano plots (− log₁₀ false discovery rate vs. log₂ fold change) were generated to visualize differentially expressed genes (DEGs) in the comparisons “PTPM vs. NS,” “PTPM vs. PT,” and “PTPM vs. PTM.” Principal component analysis (PCA) and hierarchical clustering assessed intergroup differences and intragroup consistency. Venn diagrams illustrated shared and group-specific differentially expressed genes.

Gene Ontology (GO) and Kyoto Encyclopedia of Genes and Genomes (KEGG) pathway analyses were performed using the *clusterProfiler* package in R (hypergeometric distribution, adjusted *p* < 0.05). (*n* = 3 per group). Enrichment outcomes were visualized as bubble and bar plots, emphasizing pathways related to keratinization and epithelialization, extracellular matrix remodeling and adhesion, angiogenesis, inflammation, and immune chemotaxis.

Transcriptomic findings were cross-referenced with histological, immunofluorescence, and molecular (qPCR/Western blot) results to construct an integrated mechanistic framework describing how PTPM hydrogels modulate the wound microenvironment and facilitate diabetic wound repair.

## Supplementary Information

Supplementary Material 1.

Supplementary Material 2.

## Data Availability

All data needed to evaluate the conclusions in the paper are present in the paper and/or the Supplementary Information.
