# Supplementary material for: Injectable ROS-scavenging and NIR-responsive nanocomposite hydrogel for staphylococcus aureus-infected diabetic wound healing
Source: J Nanobiotechnology. 2026 Mar 27;24:422. doi: 10.1186/s12951-026-04306-4 (PMC13151123; doi:10.1186/s12951-026-04306-4)
Supplement: Supplementary file 2 — Supplementary Material 2. [file 12951_2026_4306_MOESM2_ESM.docx]

Supporting Information

**Injectable ROS-Scavenging and NIR-Responsive Nanocomposite Hydrogel for Staphylococcus Aureus-Infected Diabetic Wound Healing**

**
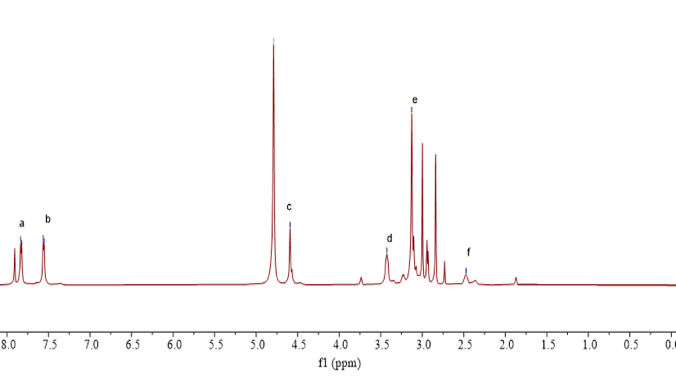
**

**Figure S1.** ¹H NMR (600 MHz, D₂O) spectrum of TSPBA

**
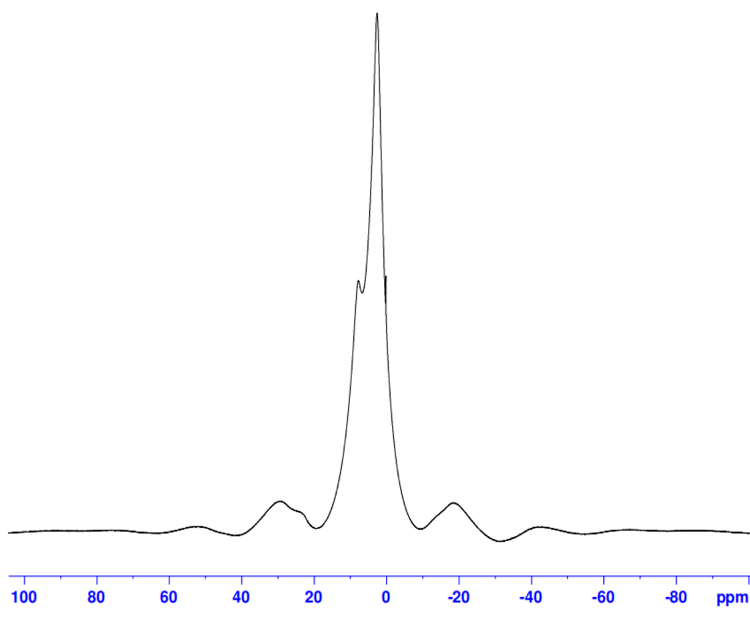
**

**Figure S2.** ¹H NMR (400 MHz) solid-state spectrum of TSPBA.

**
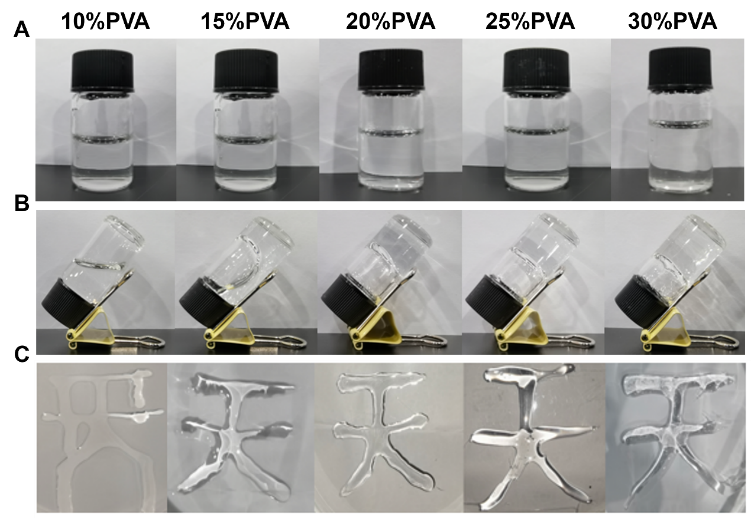
**

**Figure S3.**Gelation Process(A,B) and Injectability Evaluation(C) of PVA Hydrogels with Varying Concentrations and Ratios

**
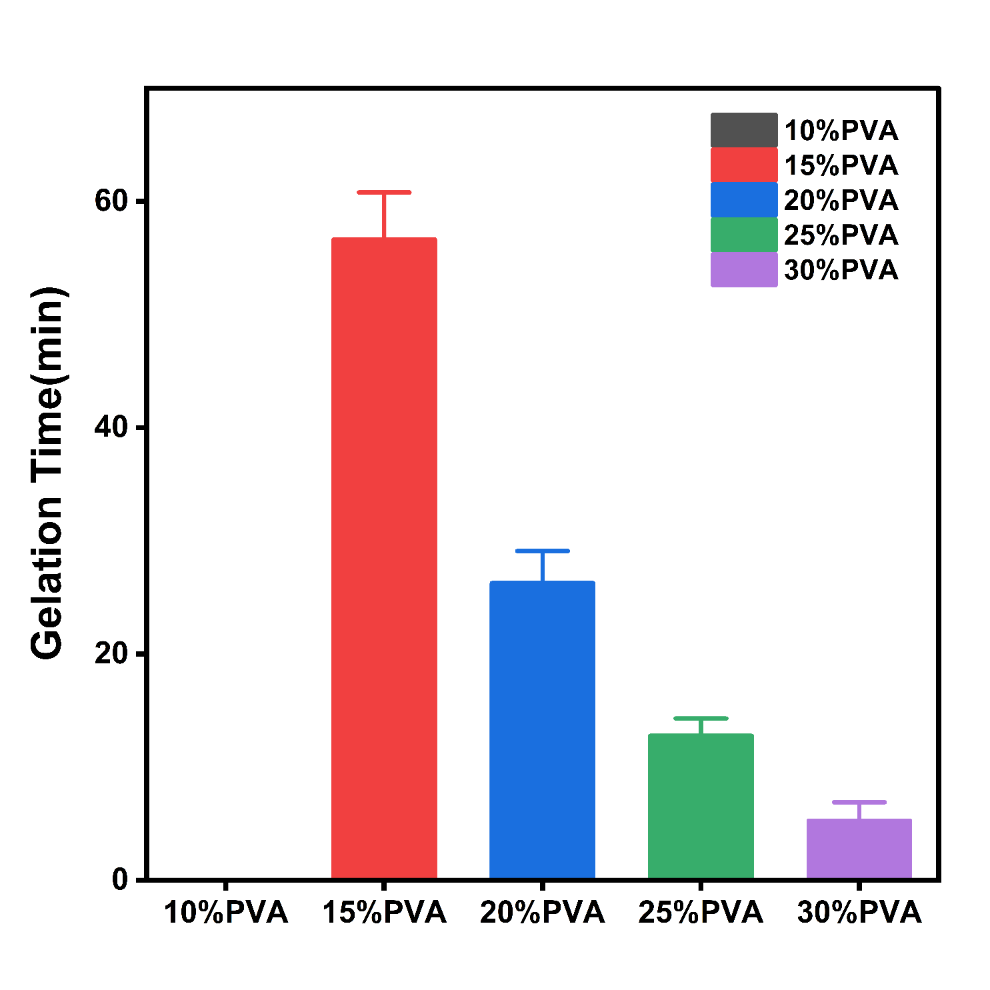
**

**Figure S4.** Gelation Time of PVA Hydrogels with Varying Concentrations and Ratios(n = 3 per group)

**
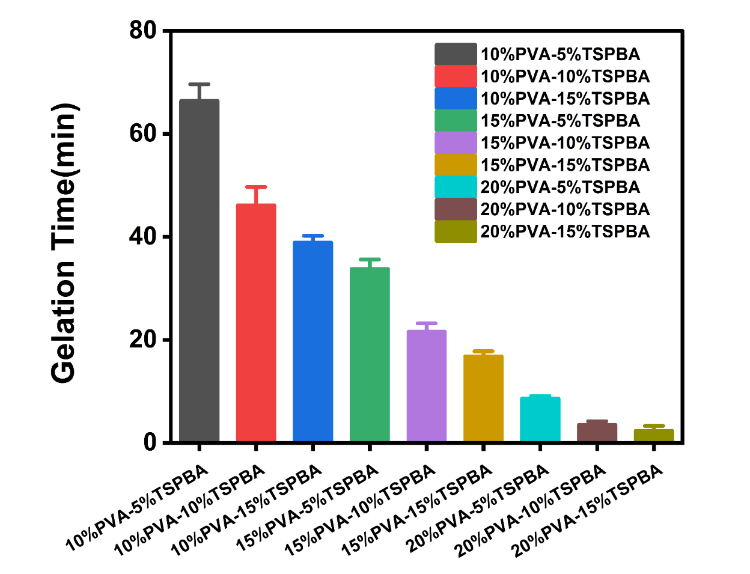
**

**Figure S5.** Gelation Time of PVA–TSPBA Hydrogels with Varying Concentrations and Ratios(n = 3 per group)

**
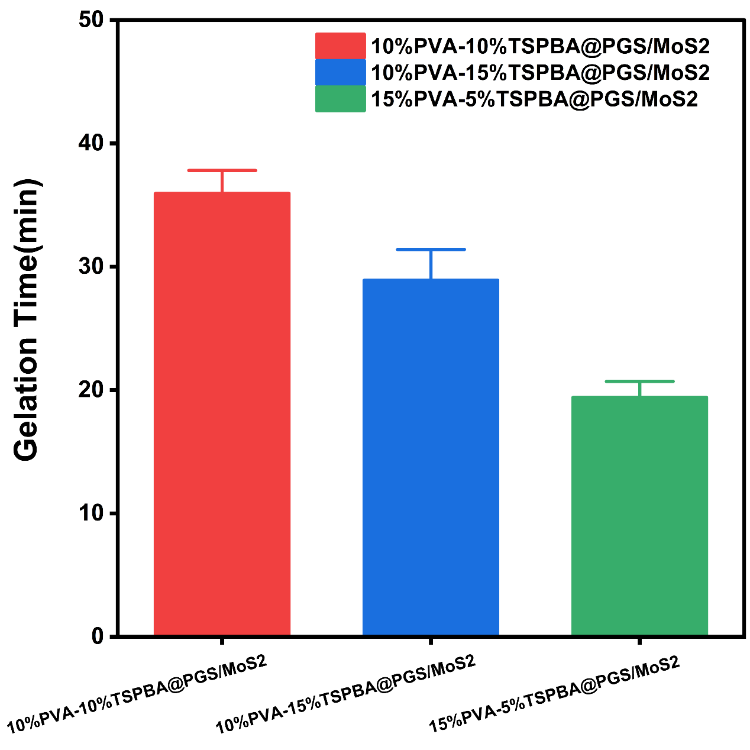
**

**Figure S6.** Gelation Time of PVA–TSPBA Hydrogels Incorporating PGS/MoS₂ Nanomaterials at Different Concentrations and Ratios(n = 3 per group)

**
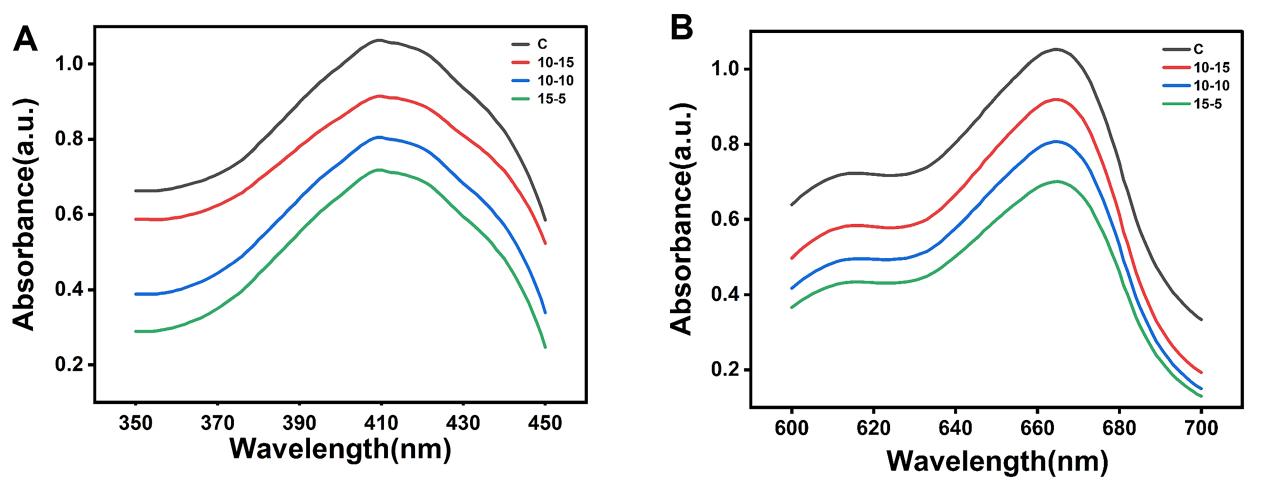
**

**Figure S7. Photodynamic evaluation showed that the 15% PVA–5% TSPBA hydrogel generated the highest ROS under NIR irradiation, as measured using DPBF(A) and MB(B).**(Group Definitions: C represents the control; 10–15 denotes the 10% PVA–15% TSPBA; 10–10 denotes the 10% PVA–10% TSPBA; and 15–5 denotes the 15% PVA–5% TSPBA)

**
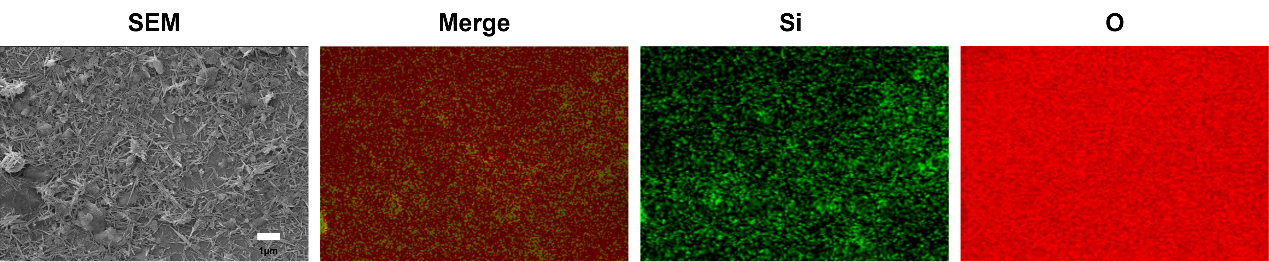
**

**Figure S8.** SEM Observation Reveals Uniform Distribution of Si and O Elements in Acid- and Heat-Treated PGS


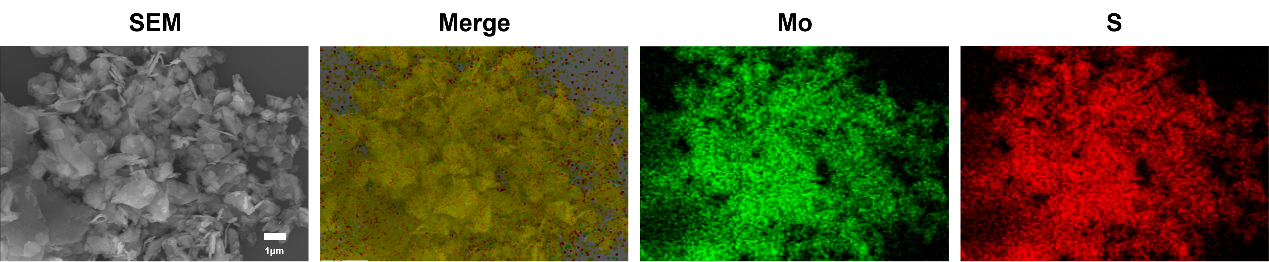


**Figure S9.** SEM Observation Reveals Uniform Distribution of Mo and S Elements in MoS₂


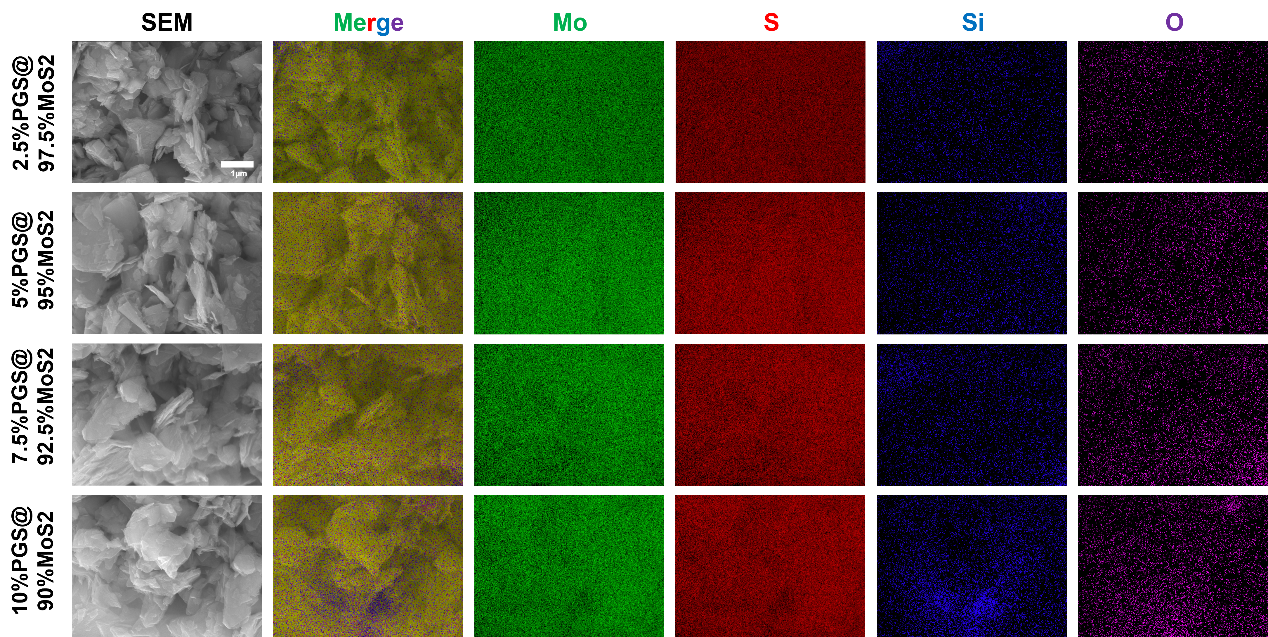


**Figure S10.** SEM Analysis of PGS/MoS₂ Nanocomposites with Varying Ratios Reveals Uniform Distribution of Mo, S, Si, and O Elements


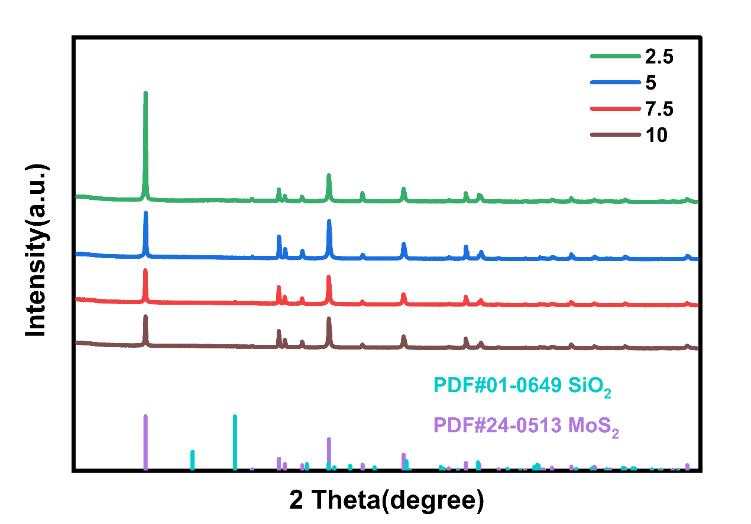


**Figure S11.** XRD Analysis of MoS₂@PGS with Varying Ratios Shows Characteristic Peaks of Major Components(2.5, 5, 7.5, and 10 represent PGS/MoS₂ nanocomposites with PGS loadings of 2.5%, 5%, 7.5%, and 10%, respectively)


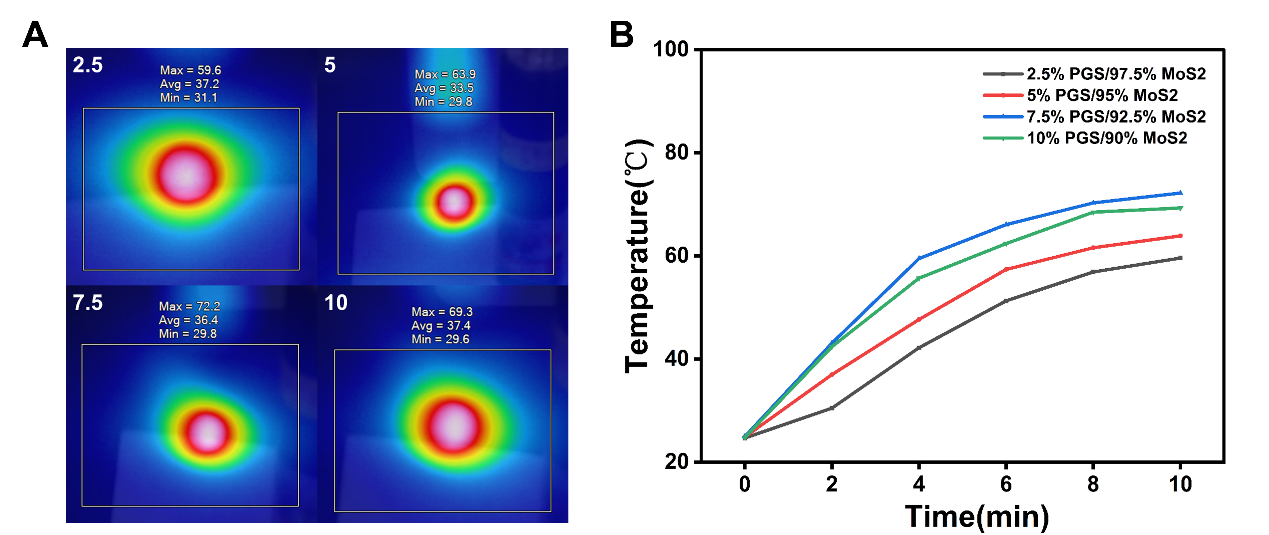


**Figure S12.** Peak Temperatures(A) and Time-Dependent Heating Profiles(B) of PGS@MoS₂ Nanocomposites under 808 nm NIR Irradiation Reveal Maximum Photothermal Conversion at 7.5% PGS/92.5% MoS₂ (2.5, 5, 7.5, and 10 represent PGS/MoS₂ nanocomposites with PGS loadings of 2.5%, 5%, 7.5%, and 10%, respectively)


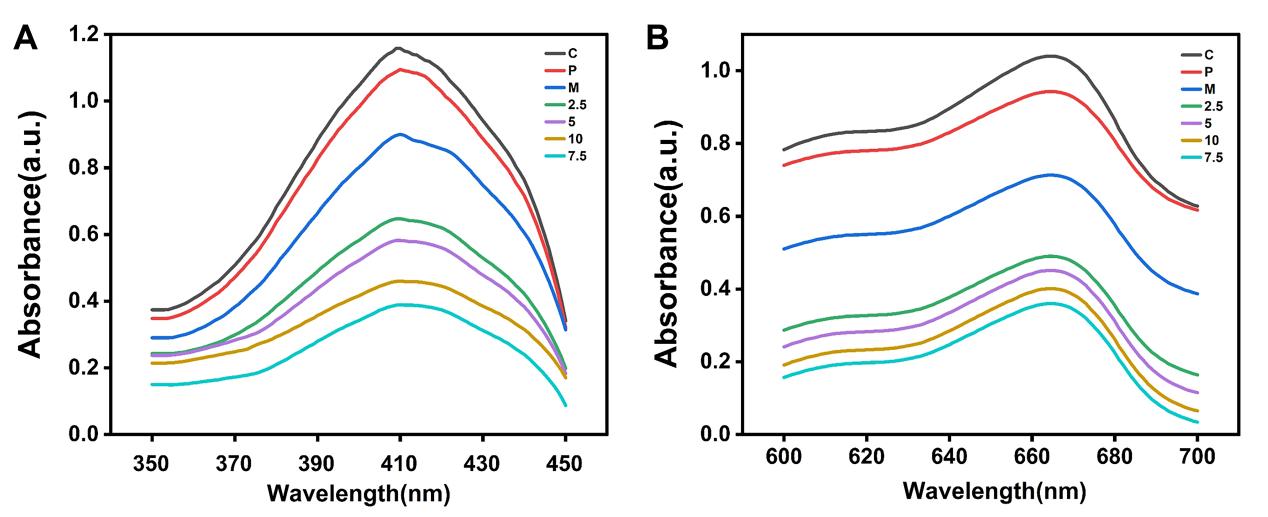


**Figure S13. ROS generation under 808 nm NIR irradiation demonstrated the optimal photodynamic performance of the 7.5% PGS/92.5% MoS₂ nanocomposites, as measured using DPBF(A) and MB(B).** (C: Control; P: PGS; M: MoS₂; 2.5, 5, 7.5, 10: PGS Loadings of 2.5%, 5%, 7.5%, and 10%, Respectively)


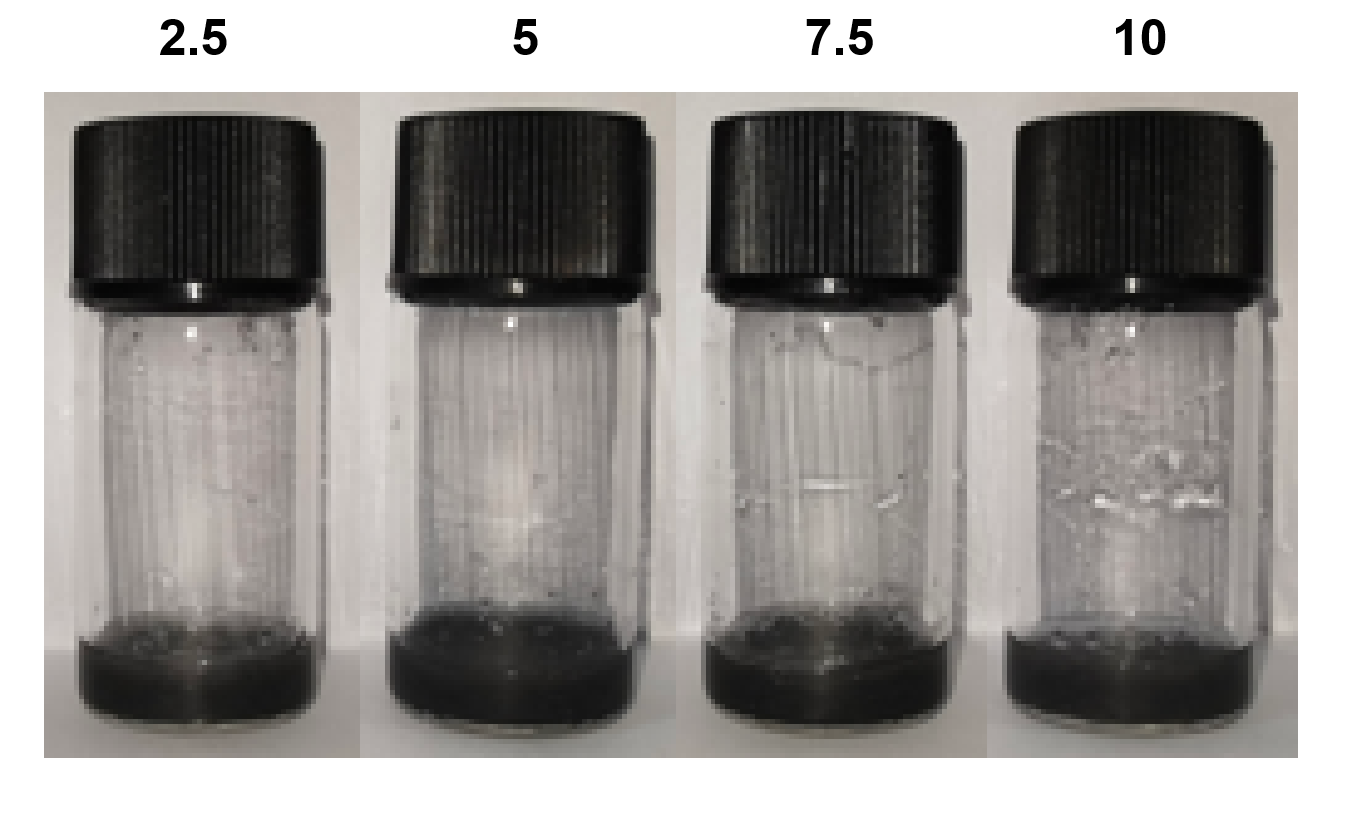


**Figure S14.** Synthesis of PVA–TSPBA Hydrogels Embedded with Nanocomposites of Varying Ratios (2.5, 5, 7.5, and 10 Represent 2.5% PGS/97.5% MoS₂@PVA–TSPBA, 5% PGS/95% MoS₂@PVA–TSPBA, 7.5% PGS/92.5% MoS₂@PVA–TSPBA, and 10% PGS/90% MoS₂@PVA–TSPBA, Respectively)


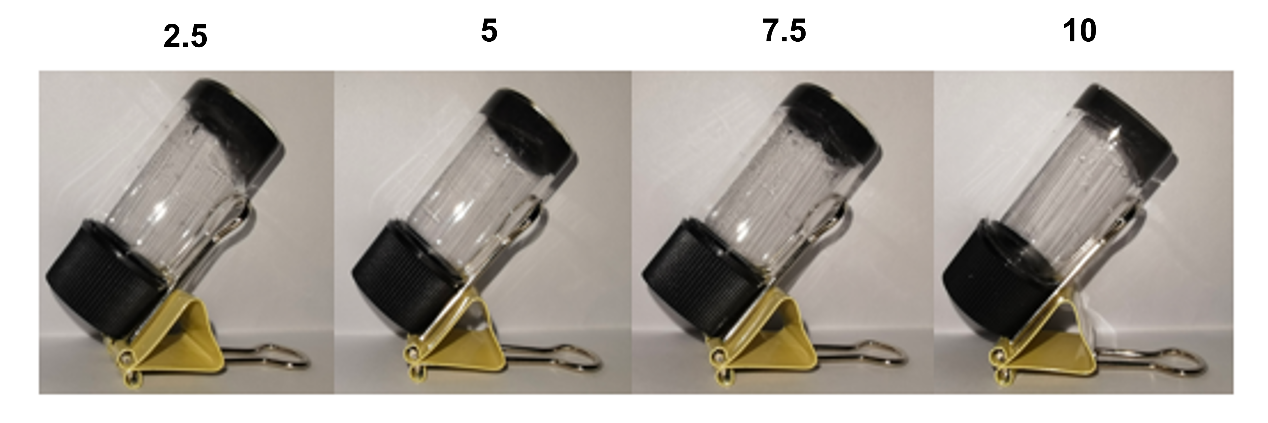


**Figure S15.** Gelation of PVA–TSPBA Hydrogels Embedded with Nanocomposites of Varying Ratios (2.5, 5, 7.5, and 10 Represent 2.5% PGS/97.5% MoS₂@PVA–TSPBA, 5% PGS/95% MoS₂@PVA–TSPBA, 7.5% PGS/92.5% MoS₂@PVA–TSPBA, and 10% PGS/90% MoS₂@PVA–TSPBA, Respectively)


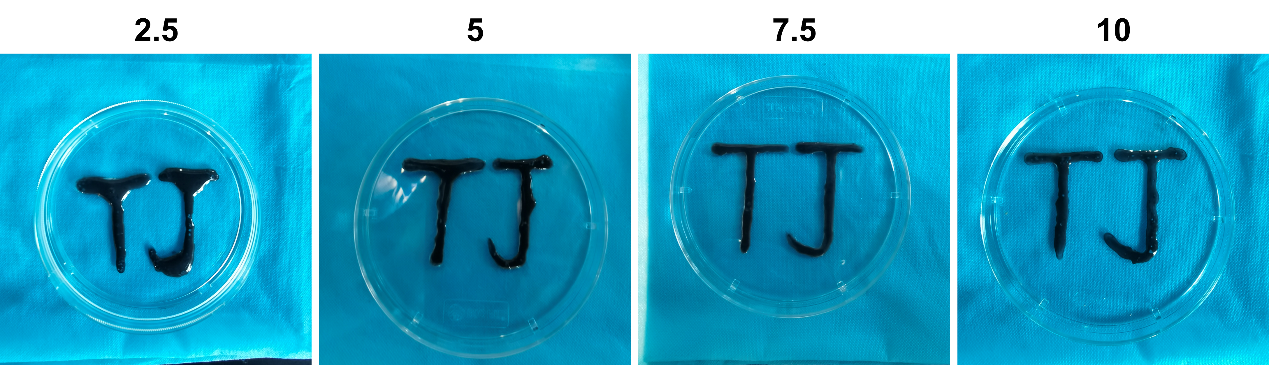


**Figure S16.** Injectability Assessment Reveals Superior Performance of 7.5% PGS/92.5% MoS₂@PVA–TSPBA Hydrogel (2.5, 5, 7.5, and 10 Represent 2.5% PGS/97.5% MoS₂@PVA–TSPBA, 5% PGS/95% MoS₂@PVA–TSPBA, 7.5% PGS/92.5% MoS₂@PVA–TSPBA, and 10% PGS/90% MoS₂@PVA–TSPBA, Respectively)


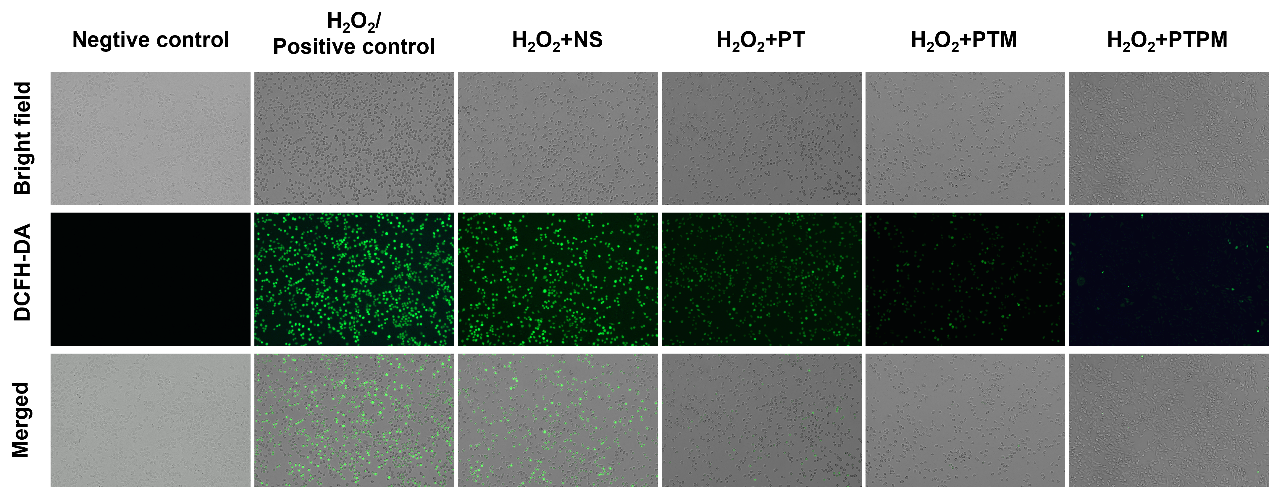


**Figure S17.** ROS Scavenging Assessment: Intracellular ROS Levels in L929 Cells Co-cultured with Hydrogels Reveal the Strongest ROS Elimination in the PTPM Group


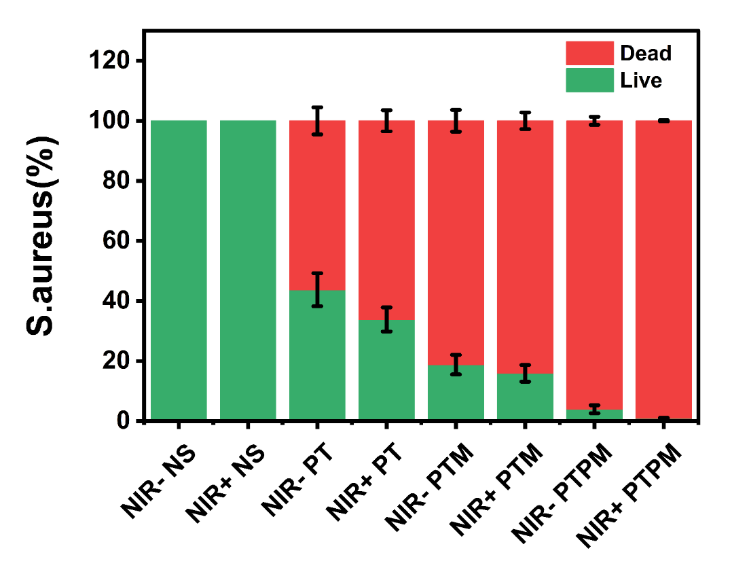


**Figure S18.** Immunofluorescence Analysis of Antibacterial Live/Dead Assay(n = 3 per group)


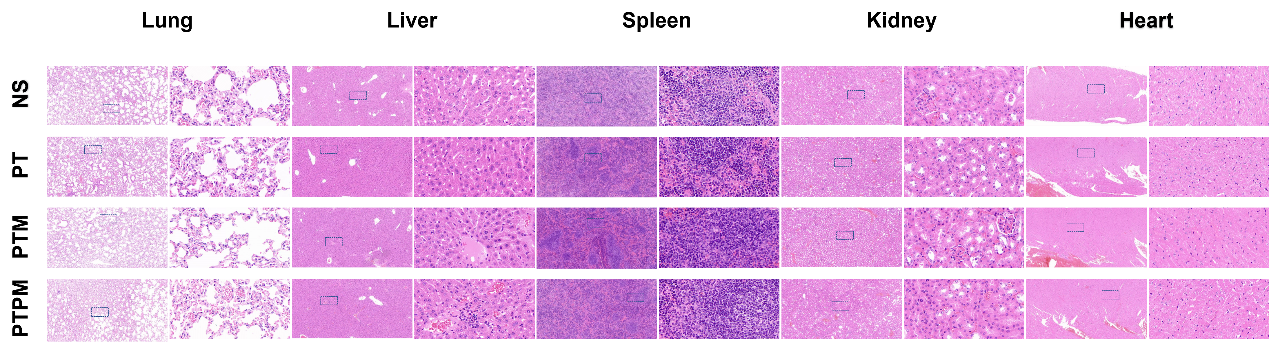
**Figure S19.** Biosafety Evaluation: Histopathological Analysis of Heart, Liver, Spleen, Lung, and Kidney in Four Groups Reveals Normal Tissue Morphology and Absence of Inflammatory or Toxic Lesions, Demonstrating Excellent Biocompatibility


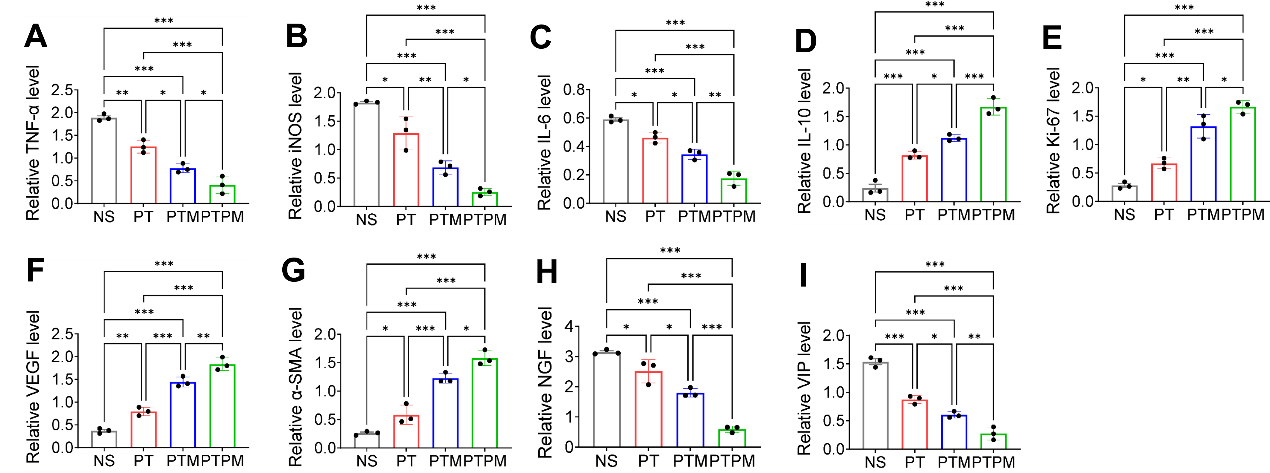
**Figure S20.** Western Blot Analysis of Inflammation- (A-D:TNF-α, iNOS, IL-6, IL-10), Epithelialization- (E:Ki67), Angiogenesis- (F-G:VEGF, α-SMA), and Neurogenesis-Related (H-I:NGF, VIP) Proteins in Wound Tissues under Different Treatments on Day 14.(n = 3 per group) Data are presented as mean ± SD; *P < 0.05, **P < 0.01, ***P < 0.001.


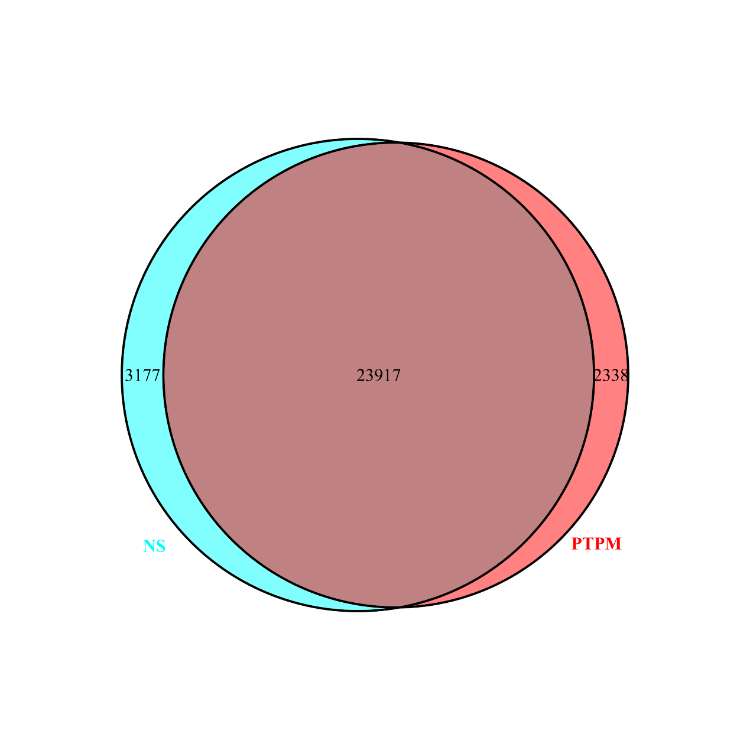


**Figure S21.** Venn diagram showing overlapping and distinct genes between PTPM and NS groups.


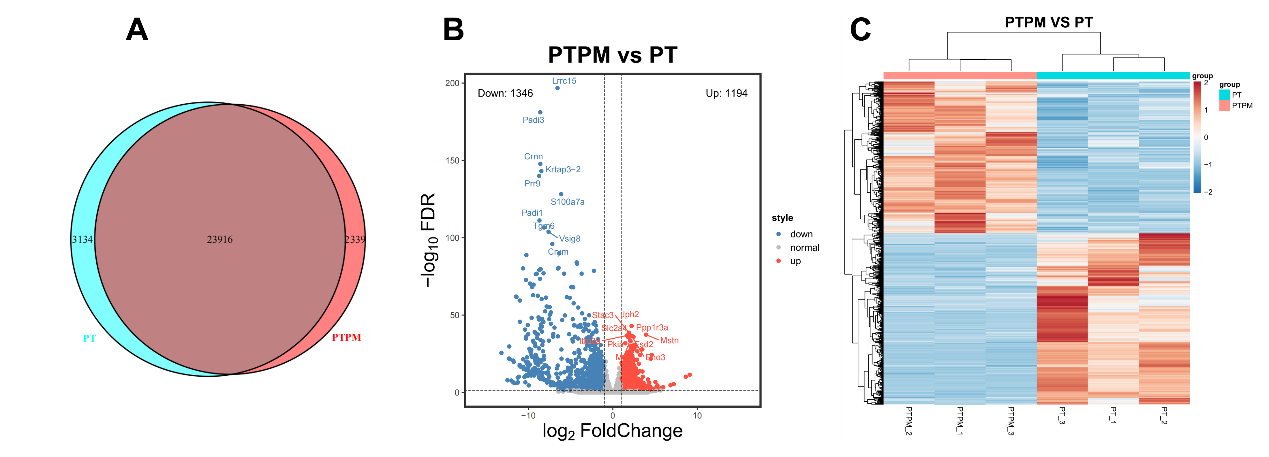
**Figure S22.** Venn diagram shows the overlapping and distinct genes between the PTPM and PT groups(A). Volcano plots illustrate the overall distribution of differentially expressed genes (DEGs) between the PTPM and PT groups, with blue and red dots representing downregulated and upregulated genes, respectively(B). Hierarchical clustering heatmap displays the similarity of gene expression patterns between PTPM and PT groups, where green and red indicate low and high expression levels, respectively, and the dendrogram reflects clustering relationships(C).


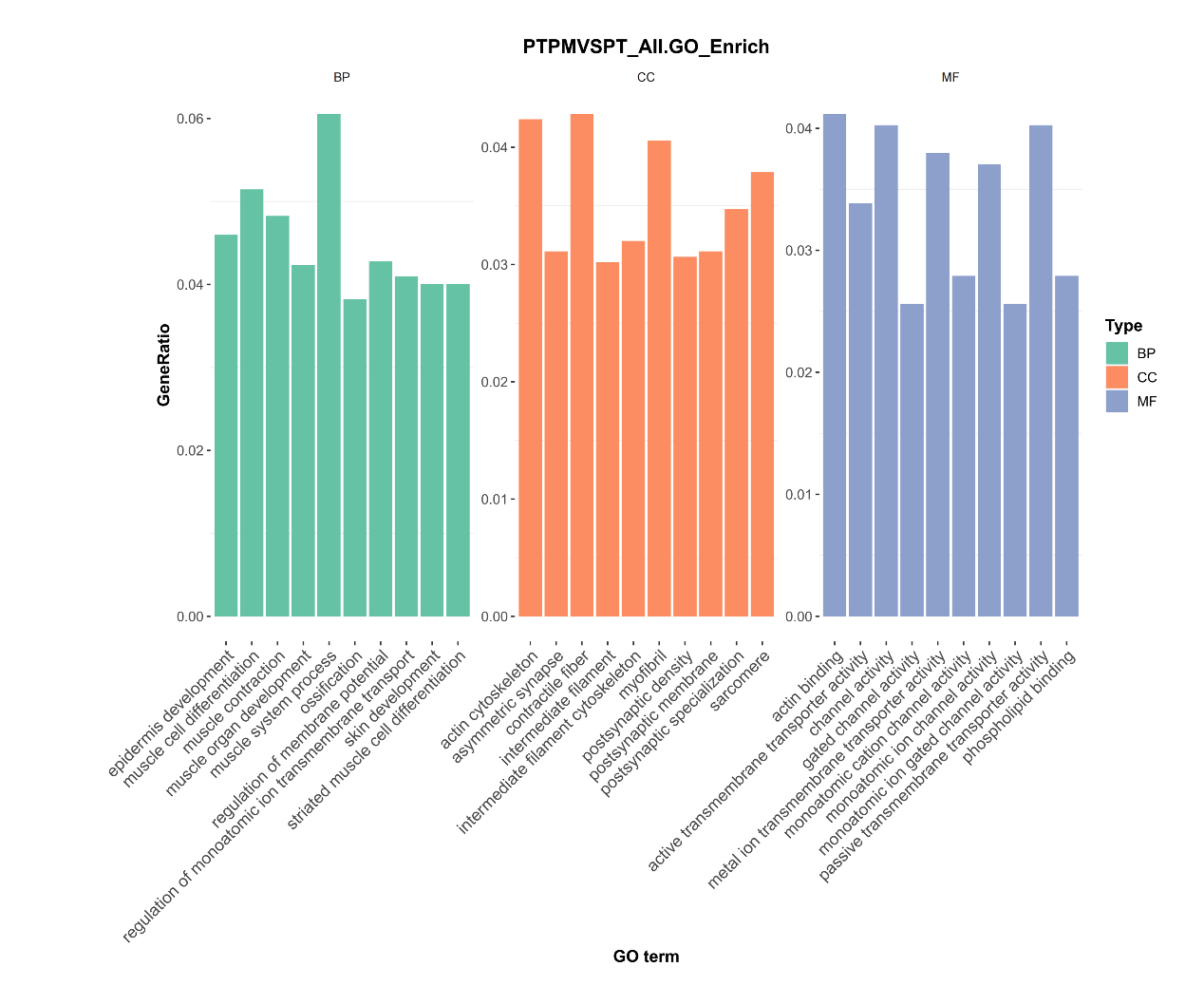
**Figure S23.** GO enrichment of DEGs in PTPM and PT groups, showing top 10 significant terms in MF ( Molecular Function ), BP( biological process ), and CC( cellular component ) categories (n = 3 per group;p.adjust < 0.05).

**
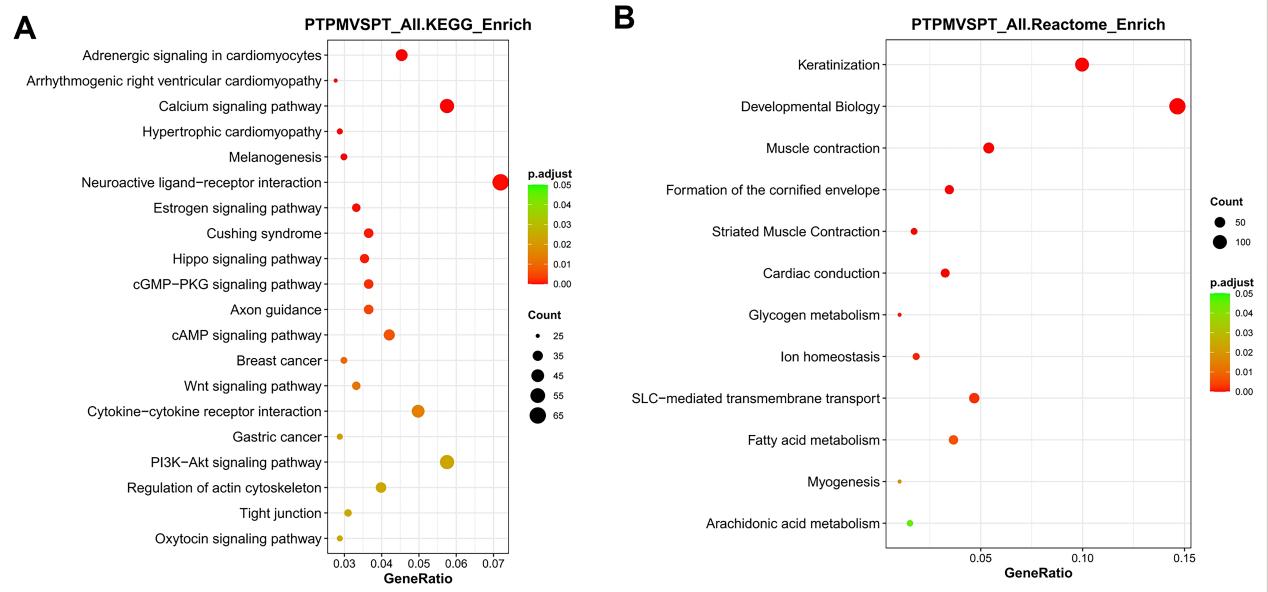
**

**Figure S24. KEGG(A) and Reactome(B) enrichment analyses of DEGs in the PTPM and PT groups, highlighting the most significant pathways.** (n = 3 per group;p.adjust < 0.05); dot size represents gene count, color indicates significance.


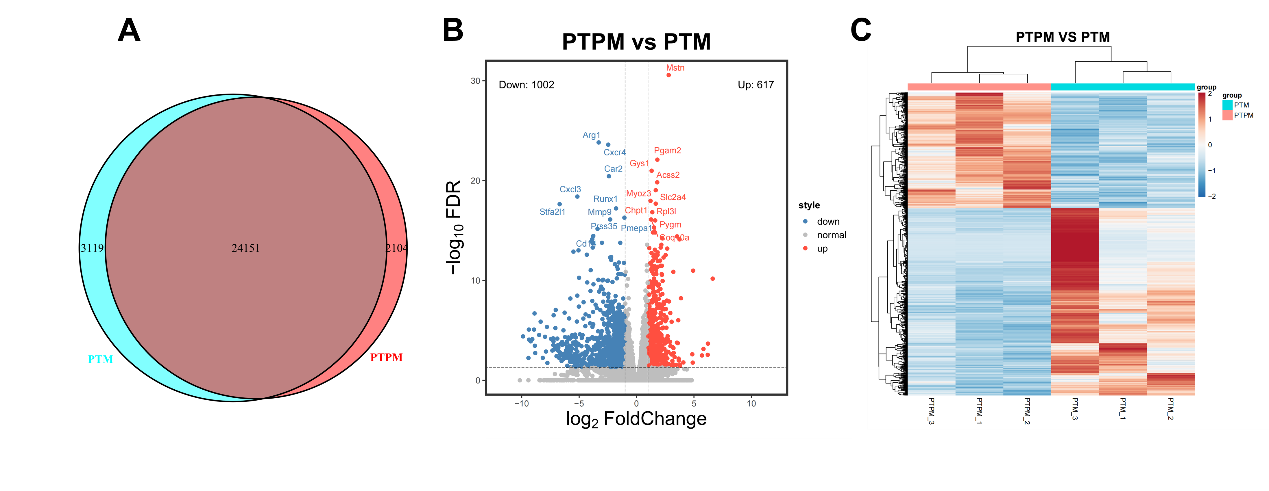
**Figure S25.** Venn diagram shows the overlapping and distinct genes between the PTPM and PTM groups(A). Volcano plots illustrate the overall distribution of differentially expressed genes (DEGs) between the PTPM and PTM groups, with blue and red dots representing downregulated and upregulated genes, respectively(B). Hierarchical clustering heatmap displays the similarity of gene expression patterns between PTPM and PTM groups, where green and red indicate low and high expression levels, respectively, and the dendrogram reflects clustering relationships(C).


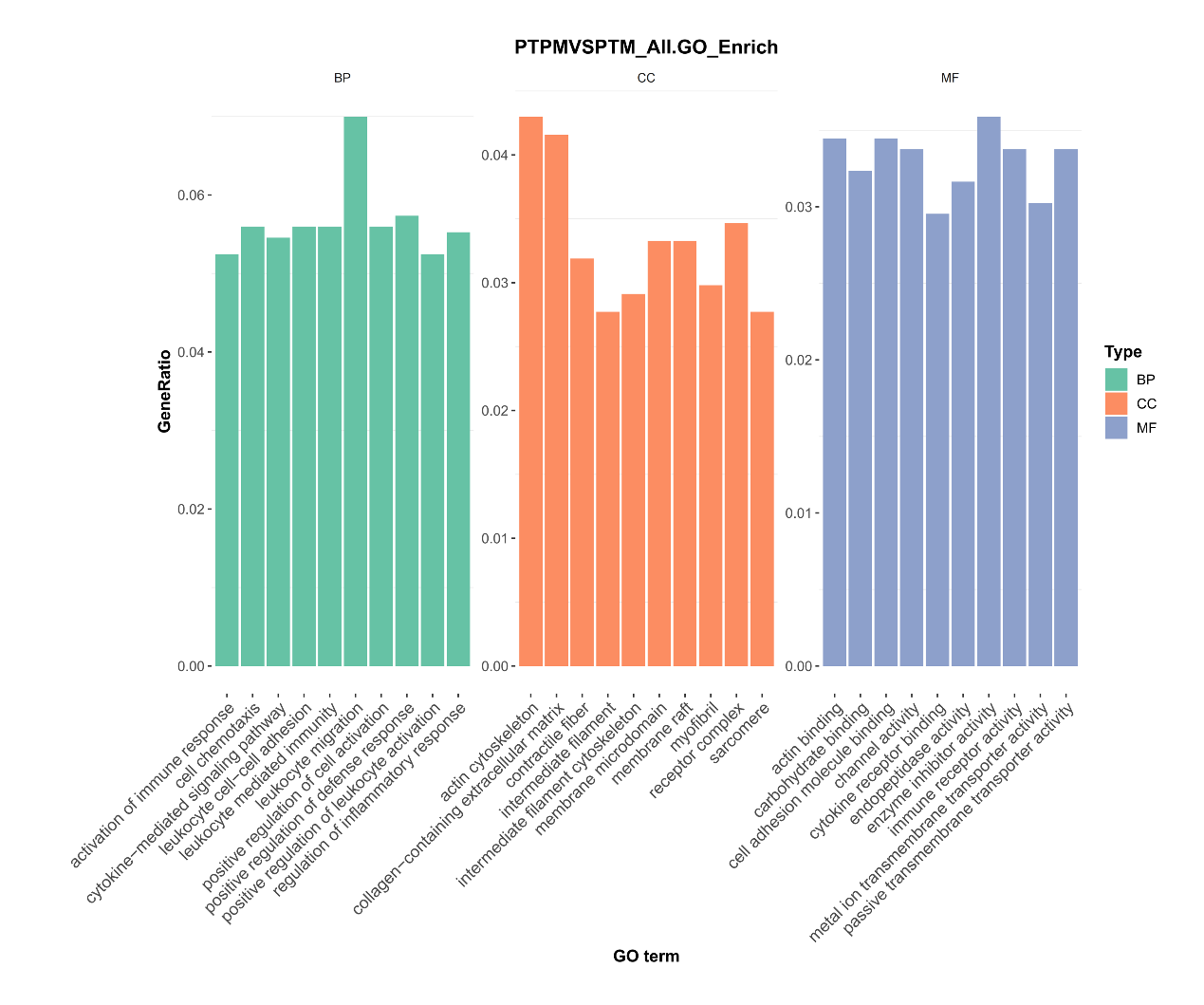
**Figure S26.** GO enrichment of DEGs in PTPM and PTM groups, showing top 10 significant terms in MF ( Molecular Function ), BP( biological process ), and CC( cellular component ) categories (n = 3 per group;p.adjust < 0.05).

**
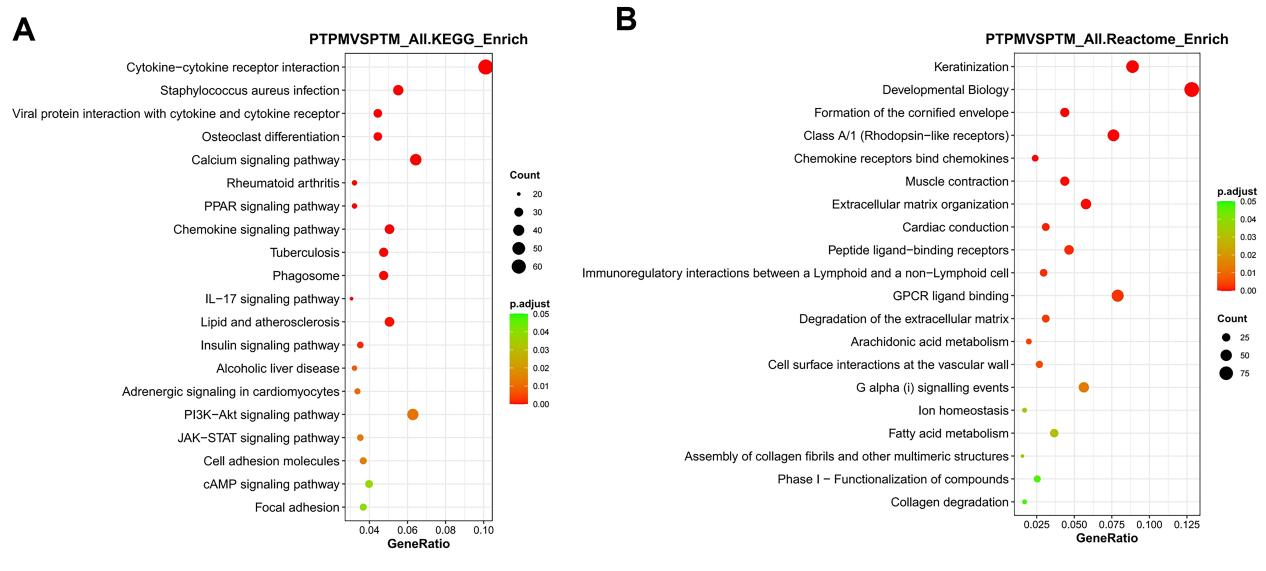
**

**Figure S27. KEGG(A) and Reactome(B) enrichment analyses of DEGs in the PTPM and PTM groups, highlighting the most significant pathways.** (n = 3 per group;p.adjust < 0.05); dot size represents gene count, color indicates significance.
